# Acridino-Diaza-20-Crown-6 Ethers: New Macrocyclic Hosts for Optochemical Metal Ion Sensing

**DOI:** 10.3390/molecules26134043

**Published:** 2021-07-01

**Authors:** Ádám Golcs, Korinna Kovács, Panna Vezse, Tünde Tóth, Péter Huszthy

**Affiliations:** 1 Department of Organic Chemistry and Technology, Budapest University of Technology and Economics, Szent Gellért tér 4., H-1111 Budapest, Hungary; korinna.kovacs11@gmail.com (K.K.); panna.vezse10@gmail.com (P.V.); toth.tunde@vbk.bme.hu (T.T.); huszthy.peter@vbk.bme.hu (P.H.); 2 Institute for Energy Security and Environmental Safety, Centre for Energy Research, Konkoly-Thege Miklós út 29-33., H-1121 Budapest, Hungary

**Keywords:** acridine, macrocycles, metal ion complexation, fluorescence, optochemical sensor

## Abstract

Acridino-diaza-20-crown-6 ether derivatives as new turn-on type fluorescent chemosensors with an excellent functionality and photophysical properties have been designed and synthesized for metal ion-selective optochemical sensing applications. Spectroscopic studies revealed that in an acetonitrile-based semi-aqueous medium, the sensor molecules exhibited a remarkable fluorescence enhancement with high sensitivity only toward Zn^2+^, Al^3+^ and Bi^3+^, among 23 different metal ions. Studies on complexation showed a great coordinating ability of log*K* > 4.7 with a 1:1 complex stoichiometry in each case. The detection limits were found to be from 59 nM to micromoles. The new ionophores enabled an optical response without being affected either by the pH in the range of 5.5–7.5, or the presence of various anions or competing metal ions. Varying the *N*-substituents of the new host-backbone provides diverse opportunities in both immobilization and practical applications without influencing the molecular recognition abilities.

## 1. Introduction

**Figure 1:**
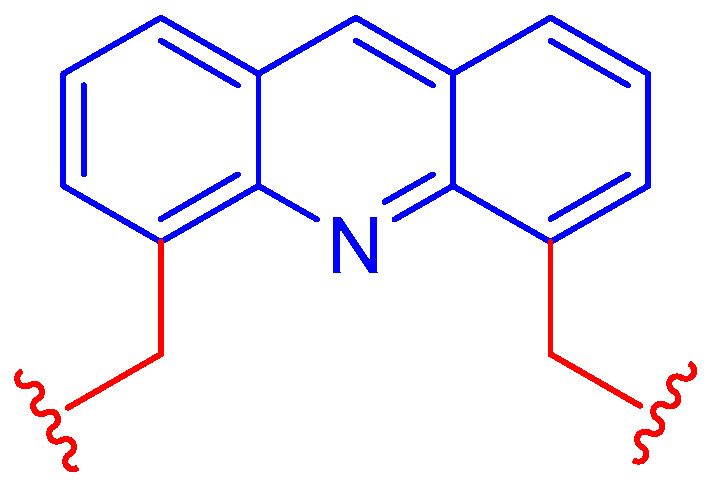
The widespread 4,5-dimethyleneacridine fluorophore unit of optochemical ionophores.

Acridine and its derivatives have been extensively studied. Researchers typically pay attention to the favorable fluorescent properties of these heterocyclic dyes. Unsurprisingly, acridines have for many years also become widespread in the field of supramolecular chemistry. They usually play an important role as optochemically active units of sensor molecules by providing the signaling function in the sensing process. In addition to the receptor unit responsible for molecular recognition, the most advanced direct-type optochemical sensor molecules also contain a signaling unit—such as an acridine fluorophore—and are able to fulfill these two basic functions of chemical sensors together [1]. They can be exemplified by a large number of recently developed optochemical ionophores containing a 4,5-dimethyleneacridine unit [2,3,4,5,6,7,8,9,10,11,12,13,14,15,16,17,18], which is shown in Figure 1.

Regarding the soft nucleophilic character of the heteroaromatic nitrogen, the application of these sensor molecules is mostly based on their selectivity for typically soft cations [2,3,4,5,6,7,8] such as Hg^2+^, Cd^2+^, Zn^2+^, Fe^3+^, Ni^2+^, but their representatives also include anion-selective ionophores [2,6,9,10,11,12,13,14,15] and host molecules selective toward various bioactive organic compounds [9,10,15,16,17,18].

As the new reported sensor molecules showed Zn^2+^-, Al^3+^- and Bi^3+^-selectivity, a brief summary of the optochemical sensor molecules with similar selectivity from the last five years is needed to put the present work into context.

Zn^2+^ plays critical roles in biology and most often acts as a cofactor in enzymes. However, its excessive intake is connected to serious disorders [19]. The detection limit (LOD) of all the recently published sensors [20,21,22,23,24,25,26] is satisfactory for the maximum permitted level of Zn^2+^ in drinking water (5 mg/L ~7.6 × 10^−5^ M [27]). The greatest challenge is to discriminate Zn^2+^ from Cd^2+^ due to their very similar chemical properties. Unfortunately, many Zn^2+^-sensitive sensor molecules do not meet this requirement [24,26]. Although the selectivity toward Zn^2+^ has been thoroughly investigated in all publications involving 11–19 potential competing cations [20,21,22,23,24,25,26], studies on the anion-coordinating ability or protonation of the ionophores as possible limiting factors of detection, did not always receive such considerable attention [20,21,22,23,24,25]. In general, the reported optochemical Zn^2+^-sensors [20,21,22,23,24,25,26] had a logarithm of their association constant (log*K*) of about 4.5–7.5 in the generally applied buffered (pH = 7.0–7.4) semi-aqueous medium (DMSO/water or MeCN/water or MeOH/water or EtOH/water).

The recently published Al^3+^-selective optochemical sensors [28,29,30,31,32,33,34,35,36,37,38,39,40] were similarly compared. Due to the similar atomic radius and valence to Mg^2+^, Ca^2+^ and Fe^3+^, Al^3+^ can act as a competitive inhibitor of these ions in biological processes [34] and can also induce neurodegenerative disorders [41]. It appears that the majority of Al^3+^-sensors show similar characteristics in operation—i.e., in the applied semi-aqueous medium, similar association constants (log*K* = 3.5–7.5) and low limits of detection (6.0 × 10^−6^ M–2.7 × 10^−8^ M), below the tolerance limit in drinking water (~7.4 × 10^−6^ M [42,43]) —as those reported for Zn^2+^-selective ones [20,21,22,23,24,25,26]. Unfortunately, the selectivity of the reported sensor molecules is often limited due to the interference of other cations, which were typically Ni^2+^ [28,31], Cu^2+^ [30,31,32,40], Hg^2+^ [30,32], Fe^2+^ [31], Mg^2+^ [36], and especially trivalent ions like Cr^3+^ [30,36,40]. In the case of Al^3+^-sensors, the study of pH-dependence is of high importance as Al^3+^ acts as a Lewis acid in water, thus adding it into a sample solution definitely results in a change of pH, which affects the photophysical behavior of the chemosensors. Nonetheless, in most cases, the study of pH-dependence does not play a major role and only an optimal pH value for operation is selected.

Unlike the previously mentioned metal ions, specific optical sensors for detecting Bi^3+^ are much rarer. As the use of bismuth in medicine has become more widespread [44,45,46], the chances of organisms to be exposed to bismuth have been increased. There are a number of instrumental techniques [47,48,49,50,51] available for its analysis; however, there are not many options for its simple chemosensor-based detection. For a few years, rhodamine, Schiff base and naphthalimide derivative-based chemiluminescent sensor molecules have been primarily used [35,52,53,54]. We note here, that to the best of our knowledge, no bismuth-selective macrocyclic host molecule has yet been reported.

In summary, strong-coordinating and regenerable hosts would be advantageous for the effective optochemical sensing of metal ions. These properties can be characterized by stability constants where, based on the relevant literature, the optimal log*K* values need to be between 3.5 and 7.5. In terms of photophysical properties and optical signaling, turn-on type ionophores with low quantum yields of free ligands are the most efficient ones, since the baseline signal of real samples is usually zero and detecting an increase in emission intensity leads to the best signal-to-noise ratio, which results in the highest sensitivity. In addition to the degree of signal change, the Stokes shift is also important. Sensor molecules, which have small Stokes shifts, restrict the sensing and imaging of their analytes in real samples. Using the sensors in physiological conditions requires a stable operation at around pH 7.0. A higher pH than 7.5 is not worth applying, as precipitation occurs in the cases of Zn^2+^, Al^3+^ and Bi^3+^, and also the biologically relevant range is up to a pH of about 7.5. In contrast, low pH can also significantly limit the applications, thus proper pH-range-tolerating sensors are in the highest demand.

Herein we report the synthesis, photophysical characterization and complexation properties of acridino-diaza-20-crown-6 ethers as a new class of fluorescent chemosensors for metal ion detection. Based on the present work, the new sensor molecules hold great promise for the future development of ion-selective optochemical sensing methods and devices.

## 2. Results and Discussion

### 2.1. Synthesis

**Scheme 1:**
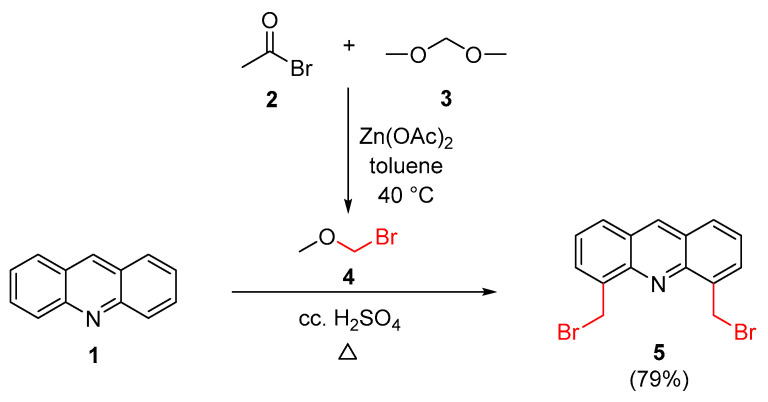
Preparation of the key intermediate 4,5-bis(bromomethyl)acridine.

For the initial step of the synthesis, the bromomethylation of acridine (**1**) at positions 4 and 5 was carried out based on a reported method [55] using bromomethyl methyl ether (BMME) as a reagent. Since, in our experience, the commercially available reagent did not have the proper quality, the alkylating agent was freshly prepared according to a previously reported procedure [56] as outlined in Scheme 1.

Bis(bromomethyl)acridine **5** is an important starting material for the subsequent synthetic steps, thus its preparation has been optimized. The yields of the reactions were examined as a function of three conditions. Experimental data can be found in Table 1.

It can be seen that raising the temperature and increasing the reaction time resulted in moderately increased yields. The optimization resulted in a 7% higher yield than that which was reported in the literature (described in [55]), after a chromatographic purification. Further studies were carried out to improve the yield by modifying the work-up procedure. When the reported chromatographic purification step [55] was omitted and the crude product was first triturated with propan-2-ol and then recrystallized twice from methanol, the yield increased to 79%. In this case, the recommended chromatographic purification was not necessary. The use of a larger excess of reagent and a temperature above 70 °C resulted in more by-products. In cases when the times of the reactions were longer than 48 h, no higher conversions were observed.

**Scheme 2:**
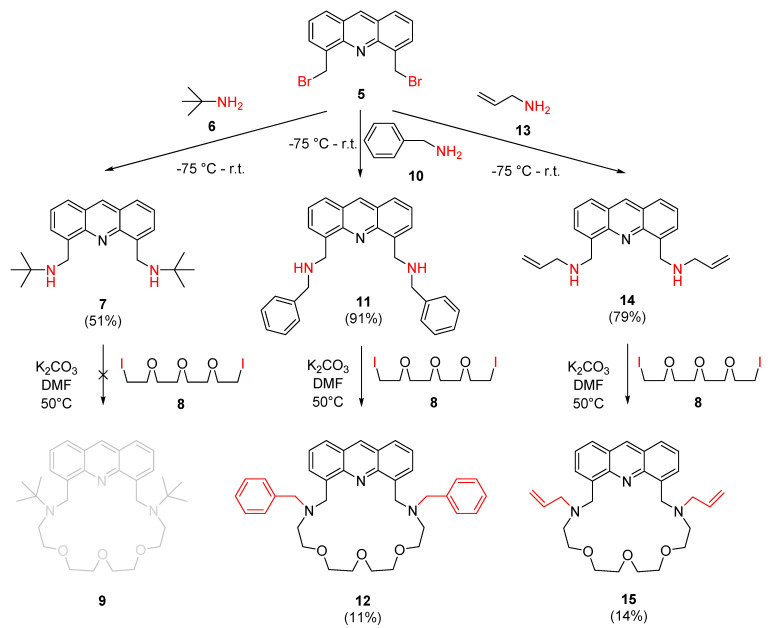
Preparation of new secondary amines (**7**, **11** and **14)** and their macrocyclization with tetraethylene glycol diiodide (**8**).

Bis(bromomethyl)acridine **5** was reacted with three different primary amines to prepare the new secondary amines required for the macrocyclization reactions as outlined in Scheme 2.

Diiodo-compound **8** was prepared from its dichloro-analogue according to a reported procedure [57]. It is noteworthy to mention, that diiodo-compound **8** undergoes rapid decomposition in light or above 70 °C; therefore, it is recommended that it should be stored below −30 °C and in the dark until use. The first reaction gave the expected secondary amine **7**, but the macrocyclization failed, probably due to the steric repulsion of the bulky *tert*-butyl groups. The macrocyclization with diiodide **8** and diamine **11** gave the expected macrocycle (**12**), but with a poor yield. The reaction of diamine **14** with dibromo-compound **5,** and the subsequent macrocyclization, gave a *N*-protected azacrown ether derivative **15**.

The structure of the obtained macrocycles is advantageous from several aspects. The heteroaromatic unit provides sufficient rigidity to the host to render the required selectivity, while the flexible methylene groups attached to positions 4 and 5 of the heterocycle allow a rapid conformational change, thus allowing the easy uptake of the active ion-trap conformation. Since the fluorophore unit is a part of the coordination sphere, the complexation of a guest ion induces photophysical changes directly. While the macrocycle containing benzyl groups (**12**) have a highly lipophilic character (log*P* = 6.5, log*D*_pH=7.0_ = 3.8 predicted by ChemAxon), its analogue **15** with allyl groups is suitable for direct polymerization. The former property facilitates the physical immobilization, the latter one enables the covalent immobilization of the sensor molecule, paving the way for a wide range of future applications.

**Scheme 3:**
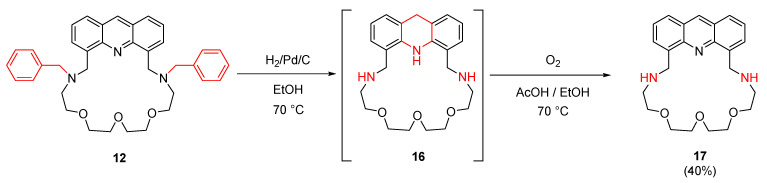
Removal of the benzyl groups to gain substitutable amine functions.

Activating the amino function of *N*-benzyl-protected macrocycle **12** was carried out by catalytic hydrogenation followed by oxidation of the unstable intermediate **16** as outlined in Scheme 3. Moreover, macrocycle **17** containing secondary amine groups can also be used as a partially water-soluble sensor molecule (log*D*_pH=7.0_ = −1.3 predicted by ChemAxon), a property which can be exploited in bioimaging.

In addition to the deprotection, the reduction of the acridine unit to acridane **16** is inevitable in these conditions. Acridane **16** was oxidized by elemental oxygen to acridine **17** in an acetic acid-ethanol mixture.

**Scheme 4:**
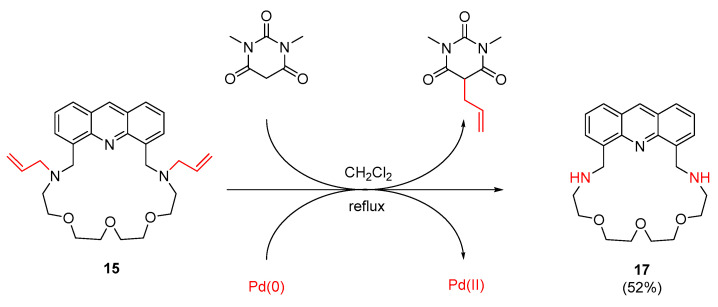
Removal of the allyl groups in an intermolecular trans-allylation reaction in mild conditions.

In the case of diallylamino-macrocycle **15**, deprotection was carried out catalytically in mild conditions based on reported analogies [58]. This is particularly important because hard conditions and acids can induce polymerization of the allyl groups. The catalyst was tetrakis(triphenylphosphine)palladium(0), which promoted the intermolecular trans-allylation reaction between the tertiary amine and 1,3-dimethylbarbituric acid via redox-processes as shown in Scheme 4.

Macrocycle **17** is a stable compound due to the benzylamine nature of its amino groups. Furthermore, it has a widely modifiable synthetic receptor backbone due to its secondary amine functionalities. This new macrocyclic derivative will hopefully serve as a precursor for a number of acridino-20-crown-6 ether-type fluorescent chemosensors.

### 2.2. Spectral Properties

**Figure 2:**
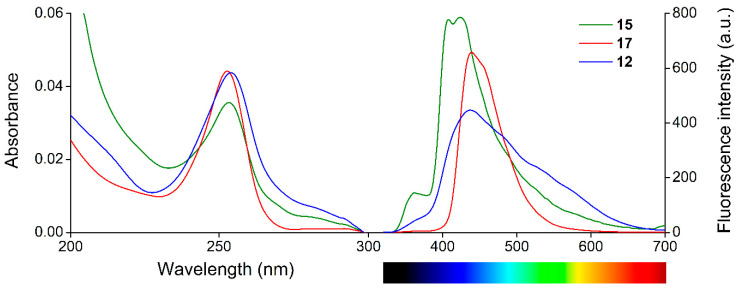
UV/Vis absorption (left) and fluorescence emission (right) spectra (λ_excitation_ = 249 nm) of the new macrocycles (c_macrocycles_ = 0.1 μM, filter = open, slit width = 10 nm) in acetonitrile.

Determination of quantum yields was carried out according to the method based on the comparison with a reference [59]. Acridine (**1**) was selected as a reference compound (Φ = 3.50 × 10^−4^ in acetonitrile [60]) and an excitation wavelength of 249 nm was chosen. The UV/Vis absorption and fluorescence emission spectra of the new crown compounds are shown in Figure 2.

The quantum yields of sensor molecules **12**, **15**, and **17** were calculated to be 2.60 × 10^−4^, 2.21 × 10^−3^, and 2.06 × 10^−3^, respectively, indicating a relatively poor fluorescence in the form of free host molecules. The absorption peak wavelengths were 253–254 nm, while the emission peak wavelengths were 424–437 nm, which resulted in extremely high Stokes shifts of 171–184 nm. This is an advantageous spectral property as it helps to eliminate autofluorescence, thus facilitates the sensitive analysis of real samples.

### 2.3. Studies on Metal Ion Complexation

**Figure 3:**
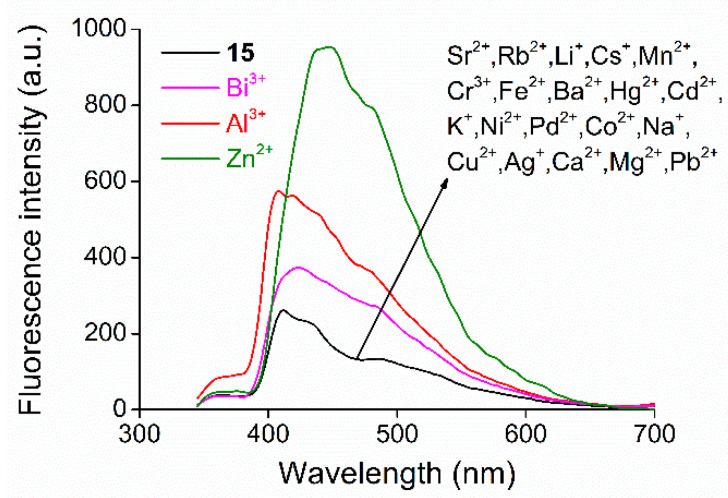
Studies on metal ion-selectivity of acridino-bis(allylamino)-macrocycle **15** (c_macrocycles_ = 1.0 μM, filter = 350 nm cut-off, slit width = 10 nm) in acetonitrile-based semi-aqueous medium.

Studies on metal ion-selectivity were carried out by adding 23 different metal salts (with carbonate counterions: Rb^2+^, Li^+^, Cs^+^; sulfate counterions: Mn^2+^, Fe^2+^; a hydroxide counterion: Ba^2+^; chloride counterions: Sr^2+^, Al^3+^, Hg^2+^, Bi^3+^; an iodide counterion: Cd^2+^; acetate counterions: K^+^, Ni^2+^, Co^2+^, Na^+^, Cu^2+^, Ag^+^, Ca^2+^, Zn^2+^, Mg^2+^, and nitrate counterions: Cr^3+^, Pd^2+^, Pb^2+^) in 50 mM aqueous solutions in 10 molar equivalent amounts (regarding the host molecule) separately to the solution of the macrocycle in acetonitrile. Among the 23 metal salts, only Zn^2+^, Al^3+^ and Bi^3+^ caused a detectable change in the fluorescence signal as shown in Figure 3.

In the cases of the other metal salts, no spectral change was observed, indicating that no complexation took place with these cations. To the best of our knowledge, macrocycle **15** is the first one to show selectivity toward Bi^3+^. Furthermore, complexed cations rarely occur as contaminants of each other in practical analysis. Although these macrocycles tend to complex three different ions, they can be considered selective in practice, as interference with competing contaminants is not expected in real samples. In the cases of macrocycles **12** and **17**, it was found that the different *N*-substituents did not affect their complexing properties (the spectra can be found in Supplementary Materials). This is not surprising as the substituents are not parts of the coordination sphere of the macrocycle. This property allows the new host analogues to be diversely *N*-substituted without altering their coordination ability, resulting in an excellent functionality of this macrocyclic backbone.

**Figure 4:**
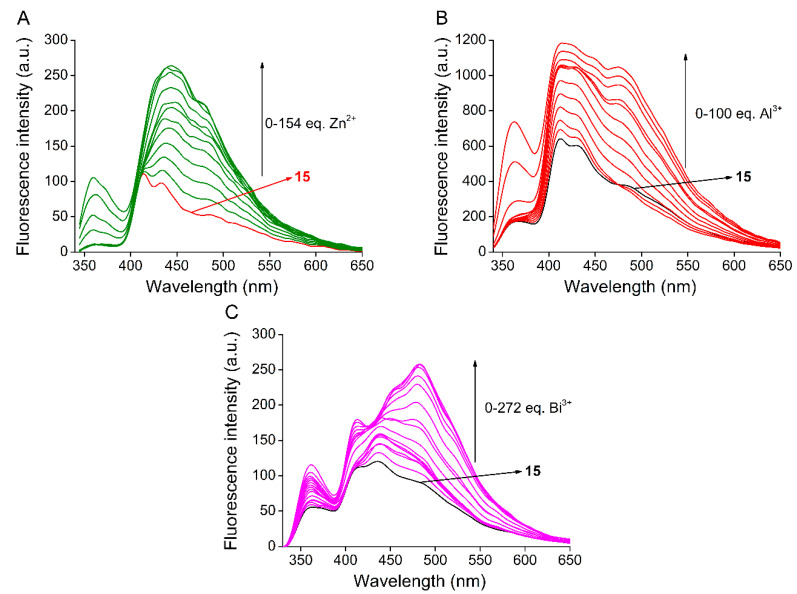
Series of fluorescence emission spectra upon titration of acridino-bis(allylamino)-macrocycle **15** with aqueous solutions of (**A**): Zn^2+^ (c_macrocycle_ = 10 μM, slit width = 5 nm), (**B**): Al^3+^ (c_macrocycle_ = 10 μM, slit width = 10 nm) and (**C**): Bi^3+^ (c_macrocycle_ = 1 μM, slit width = 7 nm) in acetonitrile (λ_excitation_ = 249 nm, filter = 290 nm cut-off).

In order to determine the stability constants (*K*) of the ligand-metal ion complexes (detailed calculation method can be found in Section 4.2) the acetonitrile solution of the host molecule was titrated with aqueous solutions of the preferred three metal salts. The observed titration series in the case of macrocycle **15** with Zn^2+^, Al^3+^ and Bi^3+^ are shown in Figure 4.

**Figure 5:**
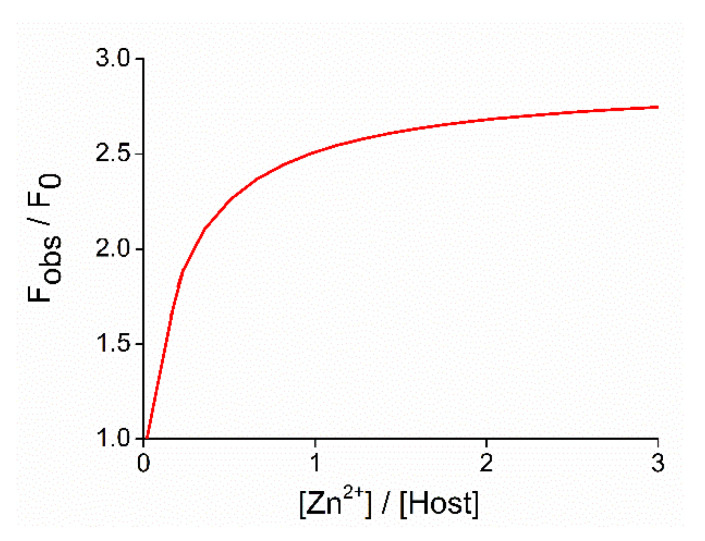
Nonlinear function globally fitted to the titration spectrum of Zn^2+^ over the entire wavelength range of measurements for calculating log*K* (F_obs_ refers to the observed fluorescence signal upon addition of the corresponding amount of Zn^2+^, while F_0_ is the initial fluorescence of free host **15**).

To determine the complex stability constants, a nonlinear regression curve was globally fitted on titration data based on the least squares method, which is shown in Figure 5 in the case of titration with Zn^2+^.

Based on the calculations, 1:1 complex stoichiometry is preferred with every metal ion. Since studies showed no significant dependence of the molecular recognition ability on the different *N*-substituents of the macrocycles, experimental results for macrocycle **15** were only reported here (further spectroscopic examinations on compounds **12** and **17** can be found in the Supplementary Materials). The calculated log*K* and LOD values are summarized in Table 2.

The results also showed that there were no significant differences among the recognition abilities of macrocycles (**12**, **15** and **17**) toward the investigated metal ions. The stability constants were above 4.7 in all cases, which indicate the formation of stable complexes among crown derivatives. However, these stability constants are not too high to inhibit the reversible operation of the sensor molecules, thus they are suitable candidates for optochemical sensing.

Reversibility and regenerability are important features for the practical application of the chemosensors. Therefore, studies on complexation were carried out several times after removing the solvents and extracting the dichloromethane solutions of the host molecules with distilled water. After regeneration, an identical enhancement of the emission intensity could be observed upon addition of 10 molar equivalents of metal salts (except in the case of macrocycle **17** due to its partial water-solubility), indicating that the new macrocycles show a reversible complexation. The regeneration could be carried out effectively by using only distilled water for extraction. No additional chelating agents, like EDTA were required for decomplexation, which makes the new sensor molecules easy to regenerate.

### 2.4. Acid–Base Properties

**Figure 6:**
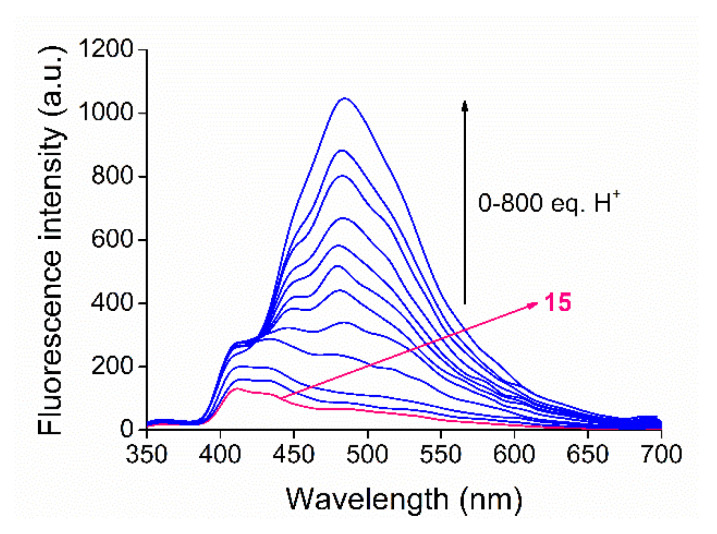
Series of emission spectra for fluorescence titration of macrocycle **15** (c_macrocycle_ = 1 μM, λ_excitation_ = 249 nm, filter = 350 nm cut-off, slit width = 5 nm) with nitric acid dissolved in acetonitrile.

In order to determine the limits of application, it is very important to study the proton association ability of the new macrocycles and the effects of protonation on spectral properties. It is known that acridine is a weak base [61] with a p*K*_a_ (conjugate acid at 20 °C) of 5.58 in water. Consequently, different ionization states are present upon acidifying the aqueous medium, which strongly influence molecular recognition. The p*K*_a_ values of the protonated aliphatic amine units of the macrocycle are 9.0 ± 0.5 in water (predicted by ChemAxon), which suggest that these nitrogens are mostly protonated in a neutral aqueous medium. The p*K*_a_ of the *N*-protonated heteroaromatic unit of macrocycle **15** was also determined in acetonitrile due to its poor solubility in water. Nitric acid dissolved in acetonitrile was gradually added to the solution of macrocycle **15**. The series of fluorescence emission spectra are shown in Figure 6.

**Figure 7:**
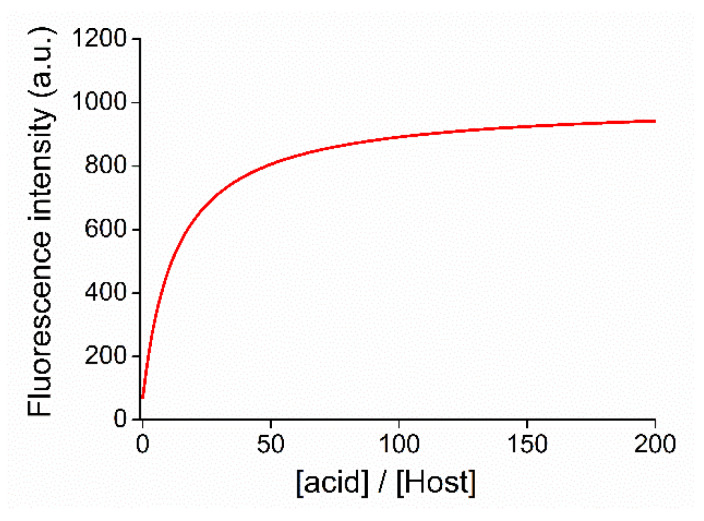
The applied global nonlinear regression curve to determine the p*K*_a_ of the *N*-protonated heteroaromatic unit of macrocycle **15**.

A molar proportional increase in fluorescence intensity was observed during the titration. A large bathochromic shift of the emission maximum indicated the appearance of a new molecular form. The p*K*_a_ was calculated according to the mathematical method detailed in Section 4.2. The applied globally fitted nonlinear regression curve is shown in Figure 7.

The calculated p*K*_a_ of the *N*-protonated acridine unit of macrocycle **15** in acetonitrile was 9.6 ± 0.1. Compared to the aqueous medium, the p*K*_a_ showed an expected increase.

**Figure 8:**
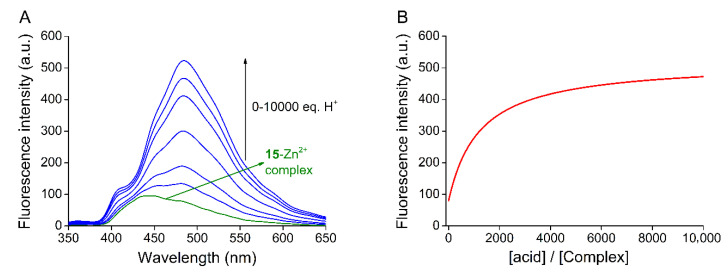
Studies on protonation of the metal ion complex by acid titration of the **15**-Zn^2+^ complex (c_complex_ = 10 μM, λ_excitation_= 249 nm, filter = 350 nm cut-off, slit width = 3 nm) with nitric acid in acetonitrile as an example (**A**) Series of fluorescence emission spectra for acid titration of the **15**-Zn^2+^ complex, (**B**) Globally fitted nonlinear regression curve for determination of p*K*_a_.

Regarding structural analogy of the new macrocycles, further studies on protonation and complexation were carried out only in the case of macrocycle **15**. Since the basicity of the complex of crown compounds differs from that of the free host, the p*K*_a_ of the protonated complex was also determined by acidic titration after adding 10 molar equivalents of Zn^2+^ to the macrocycle in acetonitrile (Figure 8).

The p*K*_a_ of the conjugate acid of the complex was 8.7 ± 0.1, which is a decrease of about one unit compared to the proton dissociation constant of the *N*-protonated acridine unit of the uncomplexed ligand, thus protonation equilibrium is suppressed by complex formation. In the complex form, no proton-induced change in emission took place in the pH range of 5.5–7.5 in acetonitrile-water mixtures, allowing the selective metal ion detection in a wide range of the biologically most relevant pH values.

It was found that the presence of protonated aliphatic amine units of the crown compound did not affect the stable coordination of cations; however, the double-positively charged parts carry potential for complexing anions. In order to study the interference of different counterions used in metal ion-selectivity studies, solutions of tetrabutylammonium salts of the corresponding anions (tetrabutylammonium cations cannot be complexed) were added to macrocycle **15** in acetonitrile.

### 2.5. Coordination Ability toward Anions

**Figure 9:**
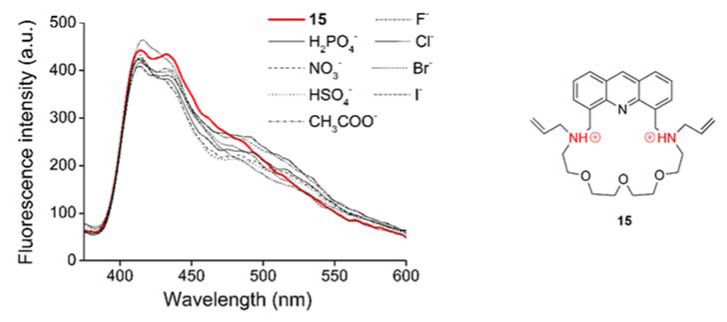
Study on the anion coordinating ability of double-protonated macrocycle **15** (c_macrocycle_ = 1 μM, λ_excitation_ = 249 nm, filter = 350 nm cut-off, slit width = 15 nm) in acetonitrile-water medium.

Various tetrabutylammonium salts (H_2_PO_4_^−^, NO_3_^−^, HSO_4_^−^, CH_3_COO^−^, F^−^, Cl^−^, Br^−^, I^−^ in 50 mM solutions) in distilled water were added to macrocycle **15** in 10 molar equivalent amounts with regard to the host molecule. No significant spectral change was observed in any of the cases (Figure 9). Thus, the double-positively charged macrocycle in a neutral water-acetonitrile medium did not coordinate the studied anions.

The reported macrocycles are weak organic bases, thus they are able to accept protons in acidic medium. Not only the double-positively charged macrocycles, but also the triple-positively charged ones can have molecular recognition abilities different from those of the corresponding neutral forms, thus studies on anion-complexation were also carried out after acidifying macrocycle **15**.

**Figure 10:**
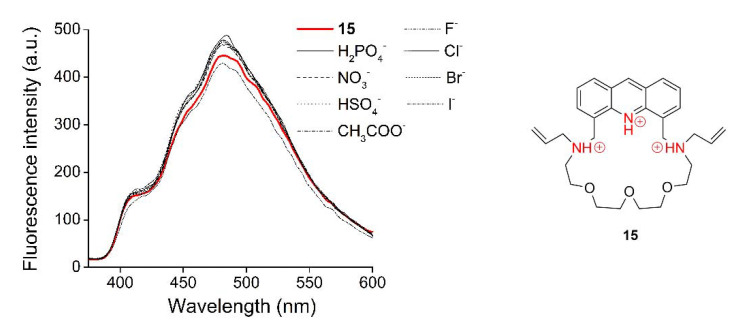
Study on the anion coordinating ability of macrocycle **15** protonated at all nitrogen atoms (c_macrocycle_ = 1 μM, λ_excitation_ = 249 nm, filter = 350 nm cut-off, slit width = 15 nm) in acetonitrile-water medium.

A solution of nitric acid in acetonitrile was added to macrocycle **15** in an amount corresponding to the end point of its acid titration (800 eq. H^+^). Then, 50 mM solutions of tetrabutylammonium salts with a 10-fold excess in distilled water, were similarly added to a solution of the macrocycle **15** protonated at all nitrogens (Figure 10).

No spectral change was observed in this case either, thus the new macrocycle does not form complexes with anions even in the fully-protonated state.

## 3. Conclusions

The parent fluorescent macrocycle (**17**) with easily functionalizable secondary amine units and its two analogues (**12** and **15**) were reported, covering the synthetic chemical background of these new hosts. The benzylated derivative (**12**) is preferred for physical immobilization, while the allyl analogue (**15**) is suitable for covalent attachment. Moreover, the improved water solubility of the parent macrocycle (**17**) can make selective imaging in living organisms possible. The new sensor molecules showed favorable spectral features—i.e., large Stokes shifts of 171–184 nm and a weak fluorescence background signal of Φ < 3 × 10^−3^ in absence of the preferred ions—for optical detection. Complexation studies were carried out with 23 metal ions. The sensor molecules showed turn-on fluorescence responses in the presence of Zn^2+^, Al^3+^ and Bi^3+^, which generally do not occur as contaminants of each other. The limits of detection were from 59 nM to micromoles for each crown derivatives. Studies on complexation showed a 1:1 complex stoichiometry and large stability constants of log*K* > 4.7 for each preferred metal ion in an acetonitrile-water medium. Reversibility and regenerability were proved by the simple and effective decomplexation and metal ion removal by extraction with distilled water. Studies showed a weak influence of the *N*-substituents on complexation, basicity and signaling. The new chemosensors did not tend to coordinate various types of anions even in their protonated forms. The p*K*_a_ of fluoroionophore **15** protonated on its acridine nitrogen atom was 9.6 in acetonitrile, which decreased about one unit upon metal ion complexation and allowed pH-independent chemosensing in the range of 5.5–7.5 in an acetonitrile-water medium. The reported new macrocycles have promising signaling properties and enable both diverse immobilization techniques and several post-synthetic modifications for future development, especially for optical sensor applications.

## 4. Experimental Methods

### 4.1. Chemicals, Apparatus and Measurements

Starting materials and reagents were purchased from Sigma-Aldrich Corporation (USA, owned by Merck, Darmstadt, Germany) and used without further purification unless otherwise noted. Solvents were dried and purified according to well established methods [62]. Silica Gel 60 F254 (Merck, Germany) and aluminum oxide 60 F254 neutral type E (Merck, Germany) plates were used for thin-layer chromatography (TLC). All reactions were monitored by TLC and visualized by UV-lamp. Aluminum oxide (neutral, activated, Brockman I) and Silica Gel 60 (70–230 mesh, Merck) were used for column chromatography. Purifications by preparative thin-layer chromatography (PTLC) were carried out using Silica gel 60 F254 (Merck, Germany) plates of 2 mm layer thickness (art No.: 1.05744) or aluminum oxide 60 F254 neutral type E (Merck, Germany) plates of 0.25 mm layer thickness (art No.: 1.05727). Ratios of solvents for the eluents are given in volumes (mL/mL). Evaporations were carried out under reduced pressure unless otherwise stated.

The new compounds were characterized by their physical constants such as melting point, thin-layer chromatography retention factor (*R*_f_), infrared, ^1^H-NMR and ^13^C-NMR spectroscopies and HRMS spectrometry. Melting points were taken on a Boetius micro-melting point apparatus and are uncorrected. Infrared spectra were recorded on a Bruker Alpha-T FT-IR spectrometer (Bruker Corporation, Billerica, MA, USA) using KBr pastilles. NMR spectra were recorded on a Bruker 300 Avance spectrometer (Bruker Corporation, USA; at 300 MHz for ^1^H and at 75.5 MHz for ^13^C spectra). HRMS analyses were carried out on a Thermo Velos Pro Orbitrap Elite (Thermo Fisher Scientific, Dreieich, Germany) system. The ionization method was ESI and was operated in positive ion mode. The protonated molecular ion peaks were fragmented by CID at a normalized collision energy of 35–45%. The samples were dissolved in methanol. Data acquisition and analysis were accomplished with Xcalibur software version 2.2 (Thermo Fisher Scientific, Germany).

UV/Vis spectra were recorded on a UNICAM UV4-100 spectrophotometer controlled by VIZION 3.4 software (ATI UNICAM, Hatley Saint George, UK). Fluorescence emission spectra were recorded on a Perkin-Elmer LS 50B luminescent spectrometer (PerkinElmer Inc., Waltham, MA, USA) and were corrected by FL Winlab 3.0 spectrometer software (PerkinElmer Inc., USA). Quartz cuvettes with a path length of 1 cm were used in all cases. Spectroscopic measurements were carried out at room temperature (25 ± 1 °C). During spectrophotometric titrations, the solutions were added with a Hamilton syringe to the acetonitrile solutions of the ligands. As macrocycles (except **17**) showed poor water solubility, their acetonitrile solutions were used for spectrophotometric studies in all cases. The reported spectra were corrected in each case with the background signal of the added solutions and concentration values were also corrected corresponding to the caused dilution.

### 4.2. Evaluation of the Results

OriginPro 8.6 (OriginLab Corp., Northampton, MA, USA) software was used for evaluation and visualization of the spectroscopic results.

Relative quantum yields were determined in acetonitrile according to a literature method [59] based on a comparison with acridine as a standard [60]. The excitation and emission spectra were recorded in the same conditions and instrument settings as in the case of the standard. The following equation was used for calculations:

(1) $$\frac{\Phi_{i}}{\Phi_{r}}=\frac{n_{i}^{2}}{n_{r}^{2}}\cdot \frac{\int_{0}^{\infty }I_{i}\left(\lambda_{ex},\lambda_{em}\right)d\lambda_{em}}{\int_{0}^{\infty }I_{r}\left(\lambda_{ex},\;\lambda_{em}\right)d\lambda_{em}}\cdot \frac{1-10^{-A_{r}\left(\lambda_{ex}\right)}}{1-10^{-A_{i}\left(\lambda_{ex}\right)}}$$

where subscript *i* refers to the sample of the initial investigated compound, while subscript *r* refers to the reference. The Φ is the quantum yield, *n* is the respective refractive index of the solvents, *I* is the fluorescence intensity, $\lambda_{ex}$ is the excitation wavelength, $\lambda_{em}$ is the emission wavelength and *A* is the absorbance.

The stability constants of the complexes were determined by global non-linear regression analysis. For determination of the complex stability constant based on the observed fluorescence enhancement upon complexation, the following equation was used [63]:

(2) $${F=I}_{0}{\Phi \varepsilon b[X]=k}_{X}\left[X\right]$$

where F is the measured fluorescence intensity, I_0_ is the intensity of the emission, Φ is the fluorescence quantum yield, ε is the molar absorption coefficient, b is the optical path length, [X] is the molar concentration of the X species and k_X_ is a constant referring to the optical properties of the X species.

In the case of complexes with 1:1 stoichiometry, the association constant can be calculated by the following equation:

(3) FF0=kHkH0+(kHGkH0)K[G]1+K[G]

where the ratios of k parameters and K were left as floating parameters during the fitting method. Parameters F and F_0_ are wavelength-dependent variables and [G] was set as a variable, too. F_0_ refers to the initial fluorescence intensity of the free host molecule, k_H_ is a constant referring to the optical properties of the free host molecule, $k_{H}^{0}$ is a constant referring to the optical properties of the free host in the presence of preferred guest molecules, constant k_HG_ describes the photophysical features of the complex, K is the association constant and [G] is the concentration of the initial guest molecules.

Global non-linear fitting was carried out similarly in the case of complexes with 1:2 (host:guest) stoichiometry based on the following equation:

(4) $$\Delta F_{\mathrm{obs}}=\frac{k_{\Delta \mathrm{HG}}{\left[H\right]}_{0}K_{1}\left[G\right]{+k}_{\Delta {\mathrm{HG}}_{2}}{\left[H\right]}_{0}K_{1}K_{2}{\left[G\right]}^{2}}{{1+K}_{1}{[G]+K}_{1}K_{2}{\left[G\right]}^{2}}$$

where ΔF_obs_ is the change in fluorescence during titration steps, k_ΔHG_ = k_HG_ − k_H_, [H]_0_ is the initial concentration of the host, K_1_ is the association constant of the first step of the complex formation equilibrium, while K_2_ is the association constant of the second step of the complexation.

The described method for the complexes with a 2:1 (host:guest) stoichiometry was carried out based on the following mathematical formula:

(5) $$\Delta F_{\mathrm{obs}}=\frac{k_{\Delta \mathrm{HG}}{\left[G\right]}_{0}K_{1}{[H]+k}_{\Delta {\mathrm{HG}}_{2}}{\left[G\right]}_{0}K_{1}K_{2}{\left[H\right]}^{2}}{{1+K}_{1}{[H]+K}_{1}K_{2}{\left[H\right]}^{2}}$$

where [G]_0_ is the initial concentration of the guest molecule and [H] is the concentration of the free hosts. Standard errors were calculated analytically from the regression and indicated as uncertainties of the log*K* determination.

The investigations of the complex stoichiometries were also carried out by applying the described global non-linear fitting methods. These results were compared in terms of the quality of fit indicators. The choice of the model was made by a statistical *F*-probe, which aimed to test the sum of squares from each model fitting. In the cases of formulas for 1:1 stoichiometry, the observed *F*-values supported the null hypothesis that the fitted model described an appropriate relationship for characterizing the population of experimental data at a confidence level of 95%. In contrast, in the cases of models for other stoichiometries, the test implies that the applied models did not fit the experimental data. (Job plot was not used for stoichiometry estimations as reported mathematical algorithms can solve these nonlinear polynomials by exactly expressing the photophysical changes during titration experiments. Studies on every crown analogue confirm the determined 1:1 stoichiometry of complexes since the one macrocycle ring is the only coordination sphere of the hosts.)

Titration experiments were carried out with careful consideration of the relevant recommendations [64].

Limits of detection were also calculated based on the data of fluorescence titrations. To determine the signal-to-noise ratio, the fluorescence intensity of the sensor molecule was measured nine times and the standard deviation of these blank measurements was determined. Three separate measurements were carried out in the presence of the metal ions and a linear regression on the average of the measured intensities was fitted as a function of concentration of the initial metal ions to determine the slope. The limit of detection was calculated using the following equation [65]:

(6) $$LOD=\frac{3d}{s}\;$$

where *d* is the standard deviation of the optical signal of the free host and *s* is the slope of the emission intensities as a function of the concentration of the guest.

Determination of p*K*_a_ in a nonaqueous medium was carried out based on the following equation [66]:

(7) $$F=\frac{F_{max}{\left[H^{+}\right]}^{n}+F_{min}K_{acid}}{K_{acid}+{\left[H^{+}\right]}^{n}}$$

where *F* is the measured fluorescence intensity, *F_max_* is the fluorescence intensity at the starting point of acid titration, [H^+^] refers to the proton concentration, *n* shows the number of associated protons/molecules, *F_min_* is the fluorescence intensity at the end point of acid titration, *K_acid_* is the acid dissociation constant of the investigated compound. During the fitting method, the *n* and the *K_acid_* were defined as floating parameters in the equation. The value of *n* proved to be close to 1, thus it was set as a constant. Based on the known values of variable [H^+^] and wavelength-dependent variables *F*, *F_max_*, *F_min_*, parameter *K_acid_* can be determined.

In the absence of water, the spectral change is in a direct relationship with the protonation of the sensor molecule in acetonitrile, thus Equation (7) contains the concentration of H^+^ as a variable instead of the pH. During the calculation it was considered that the proton dissociation of nitric acid is strongly reduced in acetonitrile compared to the estimated total dissociation of protons in water. The p*K*_a_ for nitric acid in acetonitrile is 10.6 [67]. The [H^+^] values in Equation (7) were corrected with the degree of dissociation (*α*) corresponding to the concentration of nitric acid using the Ostwald’s dilution law:

(8) $$K_{S}=\frac{c\cdot \alpha^{2}}{1-\alpha }$$

where *c* is the corresponding molar concentration of nitric acid and *K_s_* is the dissociation constant of the acid derived from its p*K*_a_ determined in acetonitrile.

### 4.3. Synthesis of the New Compounds

#### 4.3.1. Optimized Synthesis of 4,5-Bis(bromomethyl)acridine (**5**)

A mixture of acridine (**1**) (1.00 g, 5.58 mmol) and concentrated H_2_SO_4_ (98%, 12.5 mL) was stirred under argon at room temperature and BMME (**4**) (3.04 g, 22.32 mmol) was added to it in one portion. The temperature of the mixture was raised to 65 °C, when intense formation of reddish-brown gas was observed. The mixture was maintained at this temperature for 48 h, then it was poured onto 300 g of crushed ice and stirred slowly for 1 h. The precipitate was filtered off and dissolved in chloroform. The organic phase was dried over MgSO_4_, filtered and the solvent was removed. The resulting yellow solid was purified by chromatography on silica gel, using a hexane/chloroform (2:1) mixture as eluent to give **5** (1.12 g, 55%) as a bright yellow powder.

When the crude product was first triturated with propan-2-ol and then recrystallized twice from methanol, the purification by chromatography was unnecessary and the yield increased to 79% (1.60 g).

*R*_f_ = 0.47 (SiO_2_ TLC, hexane/chloroform 2:1). All other physical and spectroscopic data of **5** concurred with those reported in the literature [55].

#### 4.3.2. Preparation of *N*,*N*′-(Acridine-4,5-diylbis(methylene))bis(2-methylpropan-2-amine) (**7**)

Freshly distilled *tert*-butyl amine (29 mL, 274 mmol) was added dropwise to 4,5-bis(bromomethyl)acridine **5** (500 mg, 1.37 mmol) under an argon atmosphere at −75 °C using an acetone-dry ice cooling bath. The reaction mixture was stirred at this temperature for 1 h, then the temperature was gradually increased by 30 °C per hour while the reaction was continuously monitored by TLC. After reaching room temperature, the reaction mixture was stirred for an additional 1 h to reach complete conversion. The excess of amine was removed by distillation and the crude product was purified without any work-up procedure by column chromatography on neutral aluminum oxide using a methanol/dichloromethane (1:20) mixture as an eluent. The product was further purified by PTLC on aluminum oxide using dichloromethane as an eluent to give **7** (244 mg, 51%) as yellow crystals.

*R*_f_ = 0.28 (Al_2_O_3_ TLC, methanol/dichloromethane 1:20). M.p. = 123 °C. ^1^H-NMR (CD_3_OD): δ [ppm]: 1.49 (s, 18H); 4.84 (s, 4H); 7.67 (t, *J* = 8.2 Hz, 2H); 8.04 (d, *J* = 6.8 Hz, 2 H); 8.18 (d, *J* = 8.7 Hz, 2 H); 9.10 (s, 1 H).^13^C-NMR (CD_3_OD): δ [ppm]: 26.3; 41.7; 54.5; 125.5; 126.9; 129.0; 131.3; 133.6; 137.8; 146.4. IR: ν_max_ [cm^−1^]: 3237, 2964, 2765, 2629, 1620, 1583, 1536, 1443, 1402, 1377, 1211, 1077, 981, 895, 753. HRMS: *m*/*z* = [MH^+^]: 350.2520 (Calcd for C_23_H_31_N_3_, 349.2518).

#### 4.3.3. Preparation of 1,1′-(Acridine-4,5-diyl)bis(*N*-benzylmethanamine) (**11**)

Freshly distilled benzyl amine (30 mL, 274 mmol) was added dropwise to 4,5-bis(bromomethyl)acridine **5** (500 mg, 1.37 mmol) under an argon atmosphere at −75 °C using an acetone-dry ice cooling bath. The reaction mixture was stirred at this temperature for 1 h, then the temperature was gradually increased by 30 °C per hour while the reaction was continuously monitored by TLC. After reaching room temperature, the reaction mixture was stirred for an additional 1 h to reach complete conversion. The excess of amine was removed by distillation and the crude product was purified without any work-up procedure by column chromatography on neutral aluminum oxide using a methanol/dichloromethane (1:20) mixture as an eluent. The product was further purified by PTLC on aluminum oxide using dichloromethane as an eluent to give **11** (520 mg, 91%) as a brown viscous oil.

*R*_f_ = 0.68 (Al_2_O_3_ TLC, methanol/dichloromethane 1:10). ^1^H-NMR (CDCl_3_): δ [ppm]: 3.89 (s, 4H); 4.54 (s, 4H); 7.26 (t, *J* = 7.2 Hz, 2H); 7.33 (t, *J* = 7.5 Hz, 4H); 7.40 (d, *J* = 7.1 Hz, 4H); 7.52 (t, *J* = 8.2 Hz, 2H); 7.76 (d, *J* = 6.8 Hz, 2H); 7.95 (d, *J* = 8.7 Hz, 2H); 8.80 (s, 1H).^13^C-NMR (CDCl_3_): δ [ppm]: 51.0; 53.4; 125.6; 126.6; 126.9; 127.5; 128.3; 128.4; 129.6; 136.7; 140.3; 146.9; 154.0. IR: ν_max_ [cm^−1^]: 3304, 3059, 3025, 2919, 2850, 1617, 1533, 1494, 1452, 1361, 1115, 1028, 899, 754, 697. HRMS: *m*/*z* = [MH^+^]: 418.2212 (Calcd for C_29_H_27_N_3_, 417.2205).

#### 4.3.4. Preparation of *N*,*N*′-(Acridine-4,5-diylbis(methylene))bis(prop-2-en-1-amine) (**14**)

Freshly distilled allylamine (21 mL, 274 mmol) was added dropwise to 4,5-bis(bromomethyl)acridine **5** (500 mg, 1.37 mmol) under an argon atmosphere at −75 °C using an acetone-dry ice cooling bath. The reaction mixture was stirred at this temperature for 1 h, then the temperature was gradually increased by 30 °C per hour while the reaction was continuously monitored by TLC. After reaching room temperature, the reaction mixture was stirred for an additional 1 h to reach complete conversion. The excess of amine was removed by distillation and the crude product was purified without any work-up procedure by column chromatography on neutral aluminum oxide using a methanol/dichloromethane (1:50) mixture as an eluent. The product was further purified by PTLC on aluminum oxide using the same eluent to give **14** (343 mg, 79%) as a dark yellow viscous oil.

*R*_f_ = 0.52 (Al_2_O_3_ TLC, methanol/dichloromethane 1:20). ^1^H-NMR (CDCl_3_): δ [ppm]: 3.40 (d, *J* = 6.1 Hz, 4H); 4.53 (s, 4H); 5.16 (d, *J* = 10.4 Hz, 2H); 5.25 (d, *J* = 17.4 Hz, 2H); 5.99–6.07 (m, 2H); 7.51 (t, *J* = 8.2 Hz, 2H); 7.75 (d, *J* = 6.8 Hz, 2H); 7.95 (d, *J* = 8.7 Hz, 2H); 8.79 (s, 1H).^13^C-NMR (CDCl_3_): δ [ppm]: 50.8; 51.7; 116.6; 125.6; 126.7; 127.7; 130.0; 136.4; 136.8; 146.9; 153.0. IR: ν_max_ [cm^−1^]: 3300, 3072, 2963, 2901, 2816, 1670, 1641, 1534, 1449, 1260, 1093, 1017, 916, 798, 757, 707. HRMS: *m*/*z* = [MH^+^]: 318.1890 (Calcd for C_21_H_23_N_3_, 317.1892).

#### 4.3.5. Preparation of 7,19-Dibenzyl-10,13,16-trioxa-7,19,27-triazatetracyclo [23.3.1.0⁵^,**2**^**⁸**.0^21,2^⁶]nonacosa-1,3,5(28),21,23,25(29),26-heptaene (**12**)

A mixture of acridine derivative **11** (500 mg, 1.20 mmol), tetraethylene glycol diiodide **8** (596 mg, 1.44 mmol), finely powdered anhydrous potassium carbonate (1.66 g, 12.00 mmol) and dry and pure DMF (100 mL) was stirred vigorously under an argon atmosphere at room temperature for 15 min, then the temperature of the mixture was raised to 50 °C and kept at this temperature for 1 week. The solvent was removed and the residue was taken up in a mixture of dichloromethane (200 mL) and water (200 mL). The phases were shaken well and separated. The aqueous phase was extracted with dichloromethane (5 × 100 mL). The combined organic phase was shaken with saturated aqueous sodium chloride solution (1 × 100 mL). The organic phase was dried over MgSO_4_, filtered and the solvent was evaporated. The crude product was purified by column chromatography on neutral aluminum oxide, using dichloromethane as an eluent. The product was further purified by PTLC on aluminum oxide with methanol/dichloromethane (1:100) as an eluent to gain **12** (77 mg, 11%) as a brown viscous oil.

*R*_f_ = 0.23 (Al_2_O_3_ TLC, dichloromethane). ^1^H-NMR (CDCl_3_): δ [ppm]: 2.99 (t, *J* = 6.0 Hz, 4H); 3.67–3.70 (m, 4H); 3.71–3.79 (m, 8H); 3.90 (s, 4H); 4.75 (s, 4H); 7.35 (t, *J* = 7.4 Hz, 6H); 7.50–7.56 (m, 8H); 7.88 (d, *J* = 8.7 Hz, 2H); 8.72 (s, 1H). ^13^C-NMR (CDCl_3_): δ [ppm]: 53.3; 53.3; 59.3; 70.0; 70.7; 70.7; 125.7; 126.4; 126.7; 126.9; 128.4; 128.9; 136.1; 138.4; 140.5; 147.1; 162.6. IR: ν_max_ [cm^−1^]: 3060, 3026, 2864, 1676, 1452, 1385, 1297, 1242, 1113, 1028, 910, 760, 735, 698, 659. HRMS: *m*/*z* = [MH^+^]: 576.3151 (Calcd for C_37_H_41_N_3_O_3_, 575.3148).

#### 4.3.6. Preparation of 7,19-Bis(prop-2-en-1-yl)-10,13,16-trioxa-7,19,27-triazatetracyclo[2 3.3.1.0⁵^,2^⁸.0^21,2^⁶]nonacosa-1,3,5(28),21,23,25(29),26-heptaene (**15**)

A mixture of acridine derivative **14** (500 mg, 1.58 mmol), tetraethylene glycol diiodide **8** (787 mg, 1.90 mmol), finely powdered anhydrous potassium carbonate (2.18 g, 15.80 mmol) and dry and pure DMF (100 mL) was stirred vigorously under an argon atmosphere at room temperature for 15 min, then the temperature of the mixture was raised to 50 °C and kept at this temperature for 1 week. The solvent was removed and the residue was taken up in dichloromethane (200 mL) and water (200 mL). The phases were shaken well and separated. The aqueous phase was extracted with dichloromethane (5 × 100 mL). The combined organic phase was shaken with saturated aqueous sodium chloride solution (1 × 100 mL). The organic phase was dried over MgSO_4_, filtered and the solvent was evaporated. The crude product was purified by column chromatography on neutral aluminum oxide, using methanol/dichloromethane (1:100) as an eluent. The product was further purified by PTLC on aluminum oxide with dioxane/propan-2-ol (5:1) as an eluent to gain **15** (105 mg, 14%) as light brown crystals.

*R*_f_ = 0.89 (Al_2_O_3_ TLC, methanol/dichloromethane 1:10). M.p. = 74 °C. ^1^H-NMR (CDCl_3_): δ [ppm]: 2.93–2.99 (m, 4H); 3.35–3.40 (m, 12H); 3.60 (d, *J* = 6.6 Hz, 4H); 4.67 (s, 4H); 5.22 (d, *J* = 9.3 Hz, 2H); 5.30 (d, *J* = 7.5 Hz, 2H); 5.99–6.04 (m, 2H); 7.49 (t, *J* = 8.2 Hz, 2H); 7.88 (d, *J* = 8.7 Hz, 2H); 7.94 (d, *J* = 6.8 Hz, 2H); 8.72 (s, 1H). ^13^C-NMR (CDCl_3_): δ [ppm]: 53.0; 53.7; 57.6; 68.5; 70.2; 70.4; 115.7; 125.4; 126.4; 127.8; 132.3; 136.8; 136.9; 146.8; 153.0. IR: ν_max_ [cm^−1^]: 3073, 2920, 2858, 1671, 1617, 1532, 1449, 1352, 1292, 1249, 1116, 994, 918, 760. HRMS: *m*/*z* = [MH^+^]: 476.2900 (Calcd for C_29_H_37_N_3_O_3_, 475.2835).

#### 4.3.7. Preparation of 10,13,16-Trioxa-7,19,27-triazatetracyclo[2 3.3.1.0⁵^,2^⁸.0^21,2^⁶]nonacosa-1,3,5(28),21,23,25(29),26-heptaene (**17**) by the debenzylation of (**12**)

A solution of **12** (300 mg, 0.52 mmol) in ethanol (50 mL) was hydrogenated in the presence of Pd/C catalyst (90 mg, palladium/charcoal; activated, 10% Pd) at 70 °C. After 3 h the reaction was completed. The catalyst was filtered off and washed with ethanol (2 × 10 mL). The filtrate and washings were evaporated to give crude **16** as a brown amorphous product, which was used for the next step without purification.

Crude macrocycle **16** was dissolved in a mixture of ethanol (10 mL) and acetic acid (30 mL) and stirred under O_2_ at 70 °C for 2 h. The solvents were evaporated and the residue was triturated with water (50 mL), then the precipitate was filtered and taken up in water (50 mL) and dichloromethane (50 mL). The aqueous phase was extracted with dichloromethane (3 × 50 mL). The combined organic phase was dried over MgSO_4_, filtered and the solvent was removed. The crude product was purified by PTLC on aluminum oxide with dichloromethane/methanol (20:1) as an eluent, then recrystallized from ethanol to give macrocycle **17** (82 mg, 40%) as a brown solid.

*R*_f_ = 0.15 (Al_2_O_3_ TLC, methanol/dichloromethane 1:20). M.p. = 90 °C. ^1^H-NMR (CDCl_3_): δ [ppm]: 3.02–3.10 (m, 4H); 3.56–3.61 (m, 8H); 3.75–3.82 (m, 4H); 4.71 (s, 4H); 7.54 (t, *J* = 8.2 Hz, 2H); 7.80 (d, *J* = 6.8 Hz, 2H); 8.05 (d, *J* = 8.7 Hz, 2H); 8.84 (s, 1H). ^13^C-NMR (CDCl_3_): δ [ppm]: 49.8; 54.7; 69.4; 69.8; 72.1; 126.3; 126.5; 126.5; 129.9; 135.2; 150.8; 156.0. IR: ν_max_ [cm^−1^]: 3400, 2957, 2917, 2850, 1686, 1536, 1452, 1376, 1262, 1095, 909, 801, 786. HRMS: *m*/*z* = [MH^+^]: 396.2212 (Calcd for C_23_H_29_N_3_O_3_, 395.2209).

#### 4.3.8. Preparation of 10,13,16-Trioxa-7,19,27-triazatetracyclo[2 3.3.1.0⁵^,2^⁸.0^21,2^⁶]nonacosa-1,3,5(28),21,23,25(29),26-heptaene (**17**) starting from (**15**)

A solution of macrocycle **15** (300 mg, 0.63 mmol) in dry degassed dichloromethane (20 mL) was added with a syringe through a rubber septum cap to the stirred solution of the tetrakis(triphenylphosphine)palladium(0) catalyst (22 mg, 0.02 mmol) and 1,3-dimethylbarbituric acid (590 mg, 3.78 mmol) in dichloromethane (30 mL) under an argon atmosphere. The resulting solution was stirred at reflux temperature for 2 days. After cooling, the solvent was removed and replaced by ether. The ethereal solution was extracted twice with small volumes of saturated aqueous sodium carbonate to remove the unreacted barbituric acid and its mono-*C*-allyl derivative. The organic phase was dried over MgSO_4_, filtered and the solvent was removed. The crude product was purified by column chromatography on neutral aluminum oxide using methanol/dichloromethane (1:20) as an eluent to give macrocycle **17** (130 mg, 52%) as a brown solid.

Macrocycle **17** prepared this way was identical in every aspect to that reported in Section 4.3.7.

## Figures and Tables

**Table 1:** Summary of the effects of conditions on the yield of the bromomethylation of acridine.

Experiment No.	BMME (molar eq.) ^1^	Temperature (°C)	Reaction Time (h)	Yield (%)
1	4	50	18	48 [55]
2	4	50	24	49
3	4	65	24	51
4	4	65	48	55 _(chrom)_^2^_/_79 _(cryst)_^3^
5	6	65	48	37
6	4	75	48	40

^1^ Related to the amount of acridine. ^2^ Performing the reaction in optimized conditions and then purifying the crude product by column chromatography. ^3^ Performing the reaction in optimized conditions and then purifying the crude product by recrystallization.

**Table 2:** The lower limits of detection (LOD) and the logarithms of the calculated stability constants (log*K*) of the new macrocycles with the preferred metal ions.

Preferred Ion	Macrocycle	Peak Wavelength of the Complex ^1^ [nm]	LOD [M]	log*K*
Zn^2+^	**12**	444	7.0 × 10^−8^	5.5 ± 0.1
	**15**	434	1.7 × 10^−7^	5.6 ± 0.2
	**17**	446	2.3 × 10^−6^	5.1 ± 0.3
Al^3+^	**12**	437	2.2 × 10^−7^	5.5 ± 0.3
	**15**	412	5.9 × 10^−8^	5.6 ± 0.2
	**17**	419	1.9 × 10^−6^	4.9 ± 0.1
Bi^3+^	**12**	439	2.2 × 10^−7^	4.9 ± 0.3
	**15**	483	4.6 × 10^−6^	5.0 ± 0.1
	**17**	438	2.0 × 10^−7^	4.8 ± 0.1

^1^ LODs were calculated using the slope of the calibration curve recorded at the peak wavelength of the corresponding complexes according to the standard method detailed in Section 4.2.

## Supplementary Materials

The supplementary materials are available online.

## Sample Availability

Samples of the compounds are not available.
