# Supplementary material for: Acridino-Diaza-20-Crown-6 Ethers: New Macrocyclic Hosts for Optochemical Metal Ion Sensing
Source: Molecules. 2021 Jul 1;26(13):4043. doi: 10.3390/molecules26134043 (PMC8272042; doi:10.3390/molecules26134043)
Supplement: Supplementary file 1 [file molecules-26-04043-s001.zip › molecules-1253098-supplementary.pdf]

SUPPLEMENTARY MATERIALS

Acridino-diaza-20-crown-6 ethers: new macrocyclic hosts for optochemical metal ion sensing

Ádám Golcs,*[a] Korinna Kovács,[a] Panna Vezse,[a] Tünde Tóth,[a,b] Péter Huszthy[a]

[a] Department of Organic Chemistry and Technology, Budapest University of Technology and Economics, Szent Gellért tér 4., H-1111 Budapest, Hungary
[b] Institute for Energy Security and Environmental Safety, Centre for Energy Research, Konkoly-Thege Miklós út 29-33., H-1121 Budapest, Hungary
*Corresponding author. Tel.: +36 20 622 6164, E-mail address: golcs.adam@edu.bme.hu

Contents

1. Selectivity studies of macrocycles **12** and **17**

2. Determination of complex stability constants based on spectroscopic titration data

3. ^{1}H-NMR and ^{13}C-NMR spectra of the new compounds (solvent: $CDCl_3$ unless otherwise noted)

1. Selectivity studies of macrocycles 12 and 17

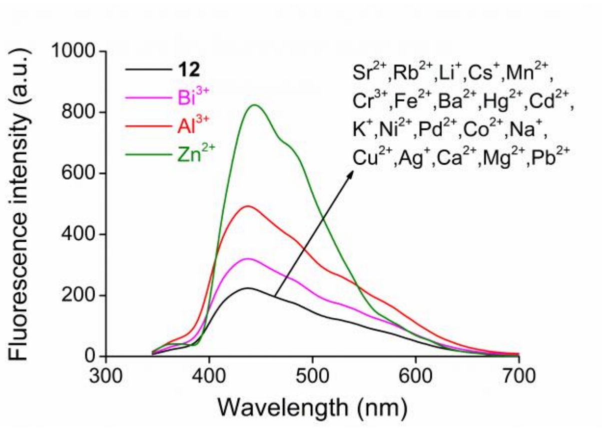

Figure S1. Fluorescence turn-on response of macrocycle **12** in the presence of 10 molar equivalents of different metal salts in acetonitrile

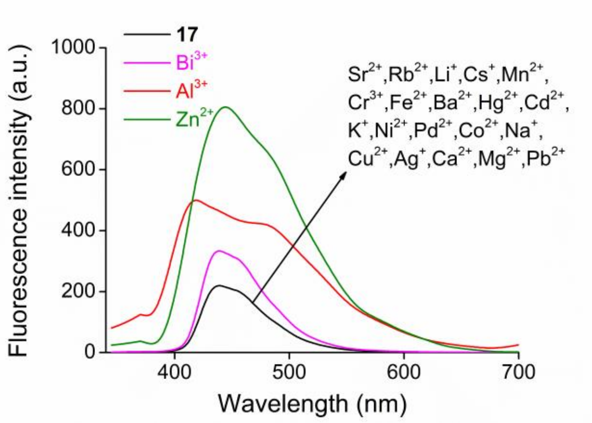

Figure S2. Fluorescence turn-on response of macrocycle **17** in the presence of 10 molar equivalents of different metal salts in acetonitrile

2. Determination of complex stability constants based on spectroscopic titration data

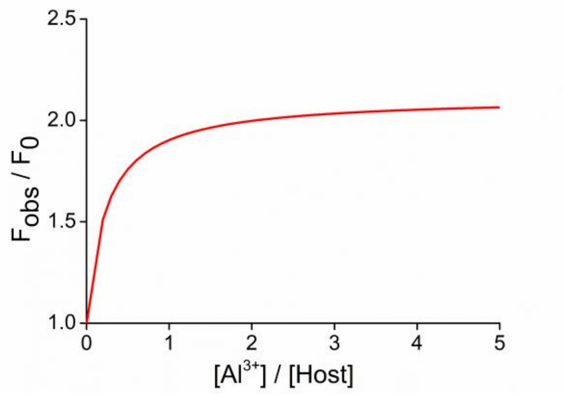

Figure S3. The globally fitted nonlinear regression curve for determining logK_a based on fluorescence titration data of macrocycle **15** with Al^{3+}

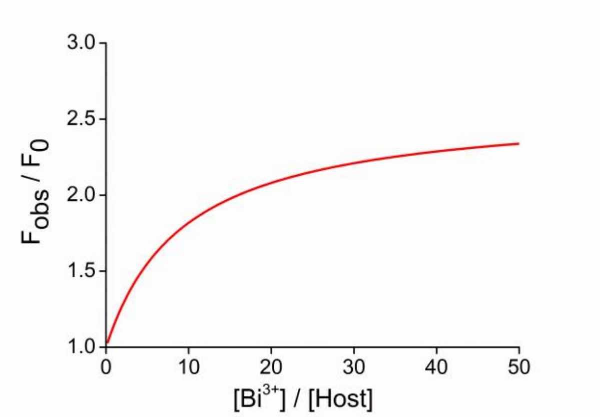

Figure S4. The globally fitted nonlinear regression curve for determining logK_a based on fluorescence titration data of macrocycle **15** with Bi^{3+}

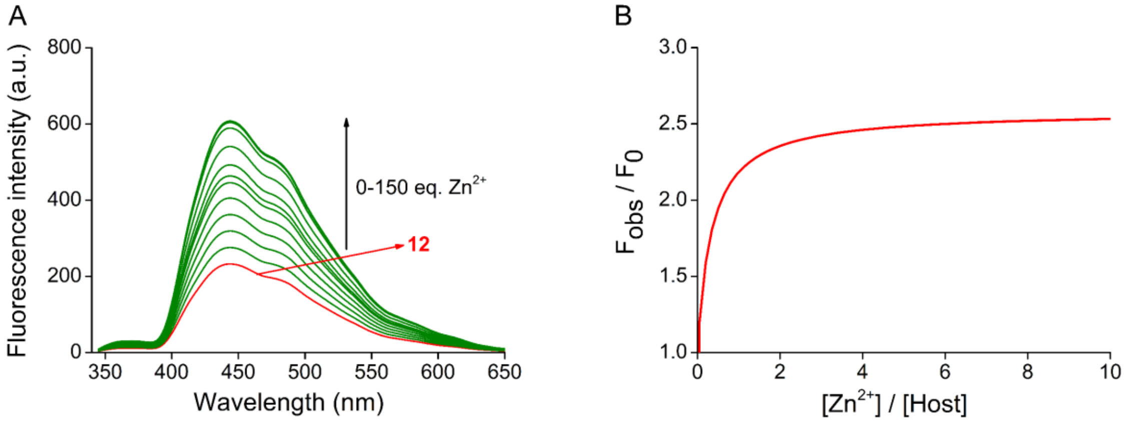

Figure S5. Series of fluorescence emission spectra for titration of macrocycle **12** with Zn^{2+} in acetonitrile (**A**) and the globally fitted nonlinear regression curve for determining logK_a (**B**)

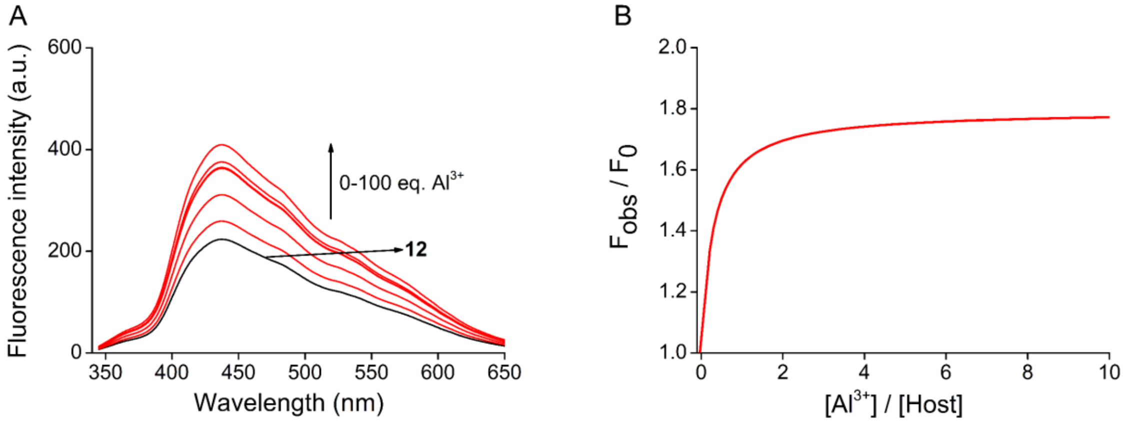

Figure S6. Series of fluorescence emission spectra for titration of macrocycle **12** with Al^{3+} in acetonitrile (**A**) and the globally fitted nonlinear regression curve for determining logK_a (**B**)

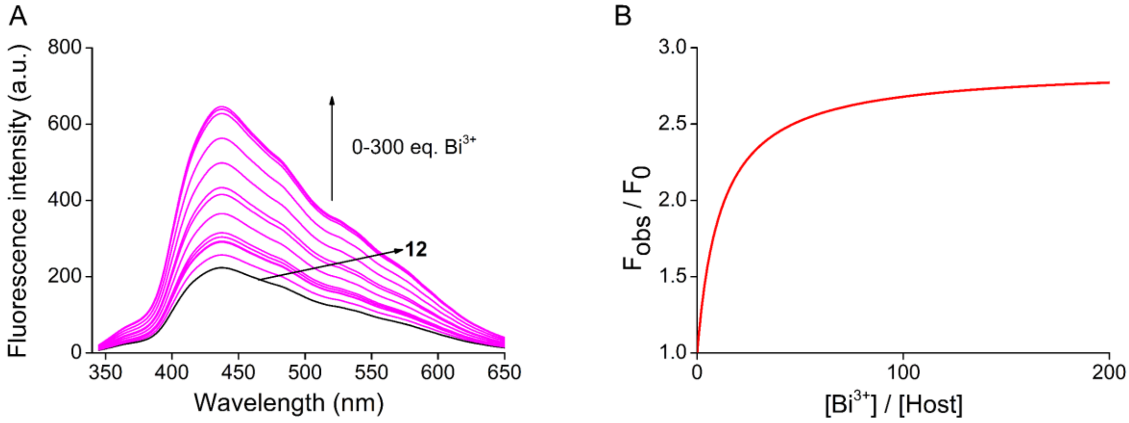

Figure S7. Series of fluorescence emission spectra for titration of macrocycle **12** with Bi^{3+} in acetonitrile (**A**) and the globally fitted nonlinear regression curve for determining logK_a (**B**)

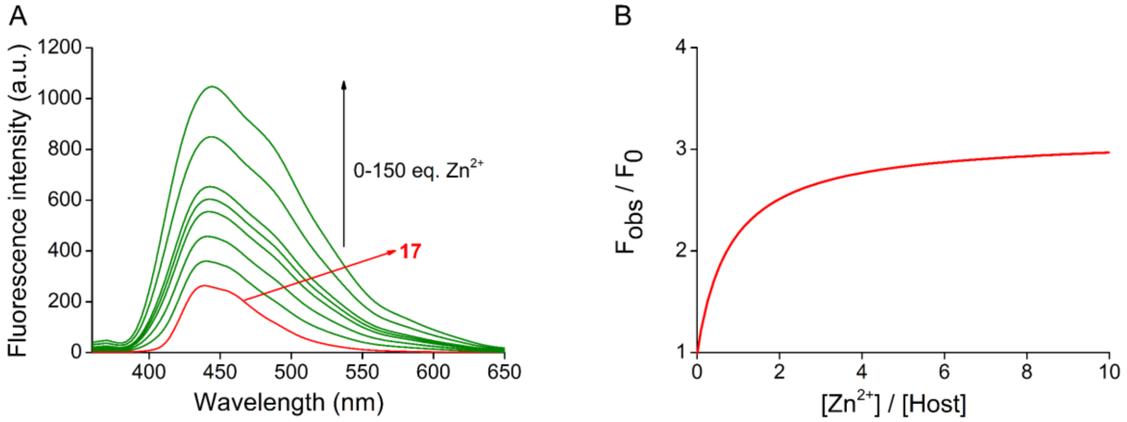

Figure S8. Series of fluorescence emission spectra for titration of macrocycle **17** with Zn^{2+} in acetonitrile (**A**) and the globally fitted nonlinear regression curve for determining logK_a (**B**)

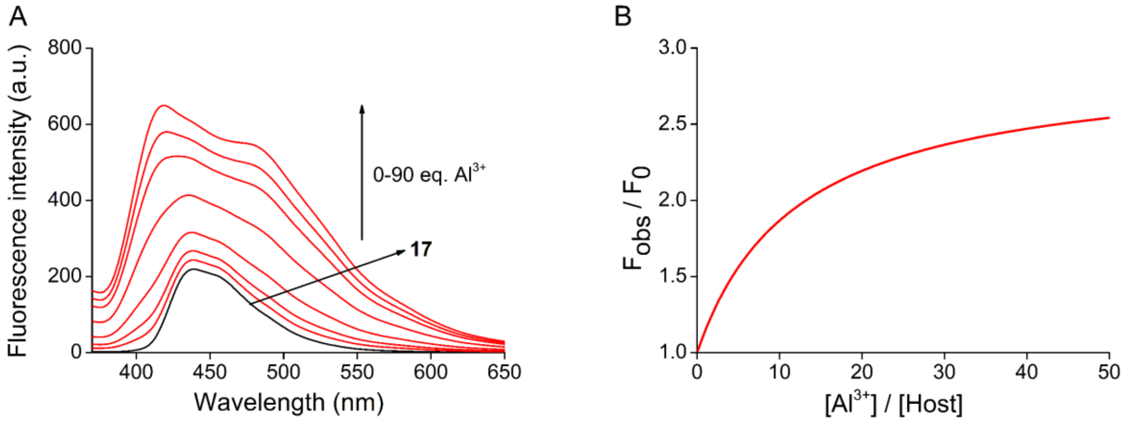

Figure S9. Series of fluorescence emission spectra for titration of macrocycle **17** with Al^{3+} in acetonitrile (**A**) and the globally fitted nonlinear regression curve for determining logK_a (**B**)

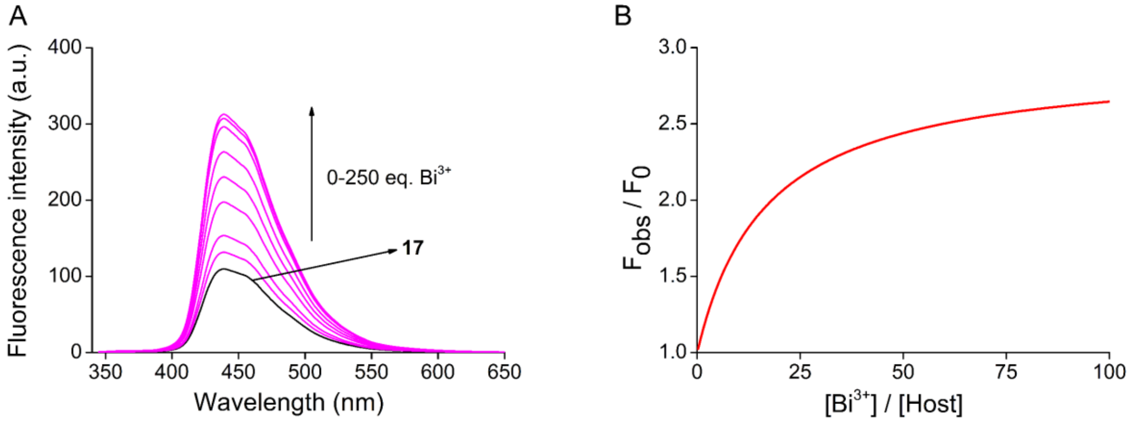

Figure S10. Series of fluorescence emission spectra for titration of macrocycle **17** with Bi^{3+} in acetonitrile (**A**) and the globally fitted nonlinear regression curve for determining logK_a (**B**)

3. ^{1}H-NMR and ^{13}C-NMR spectra of the new compounds (solvent: $CDCl_3$ unless otherwise noted)

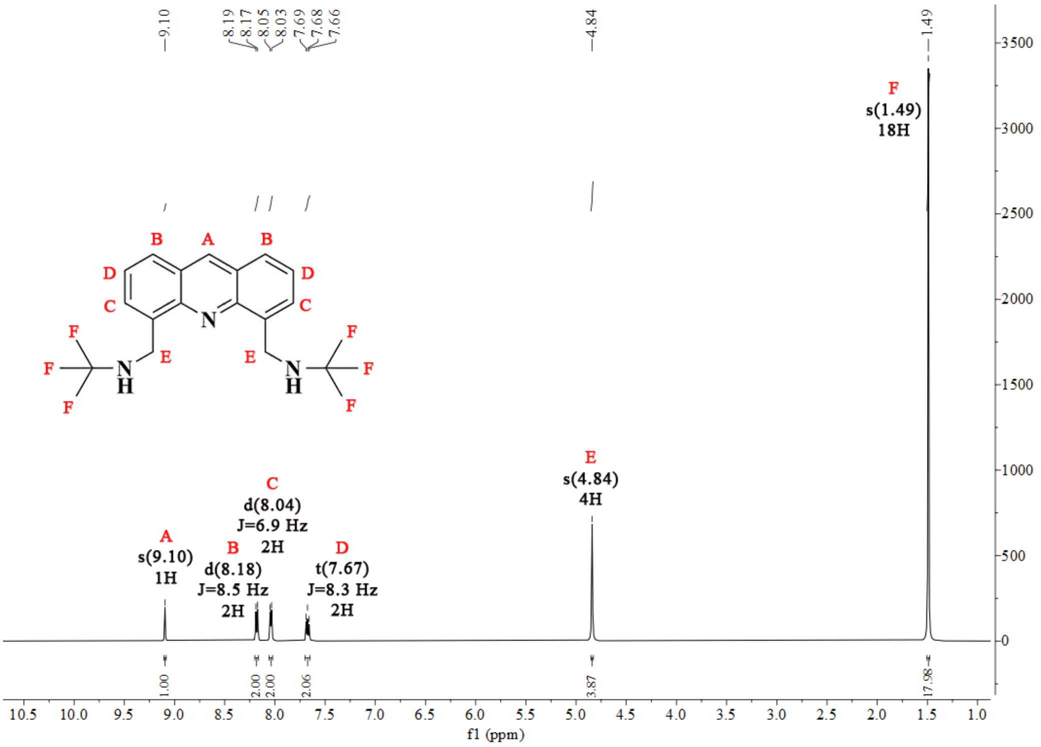

Figure S11. ^{1}H-NMR spectrum of compound **7** (solvent: CD_3OD)

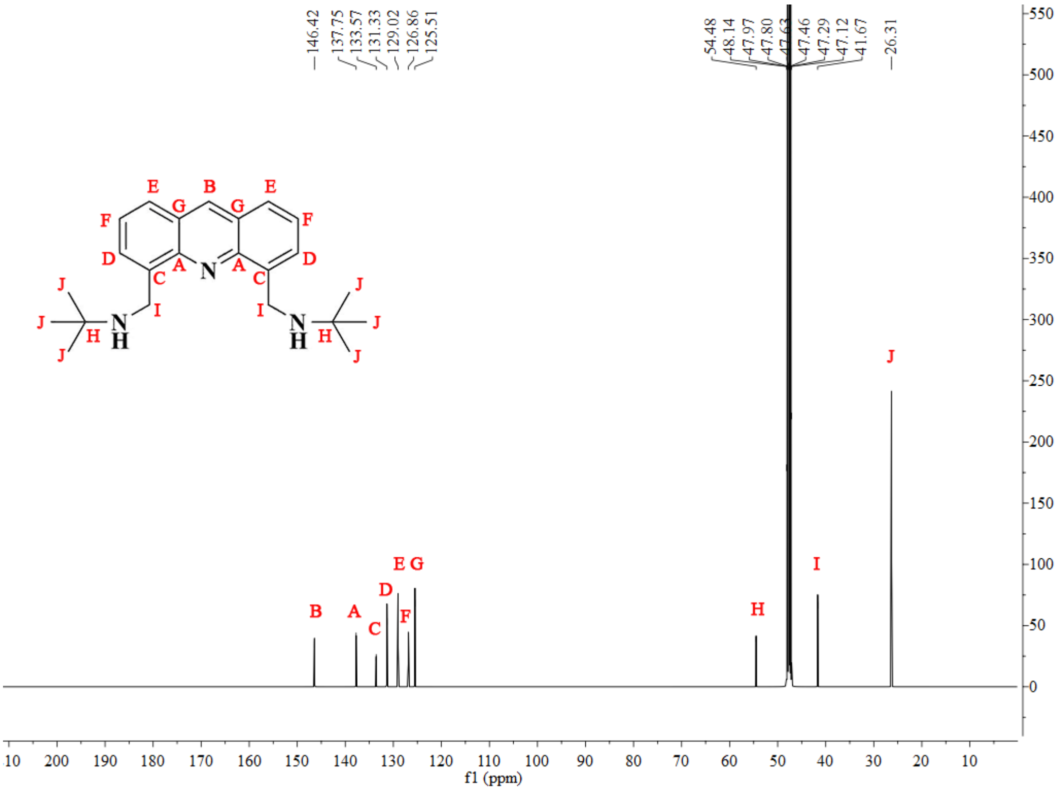

Figure S12. ^{13}C-NMR spectrum of compound **7** (solvent: CD_3OD)

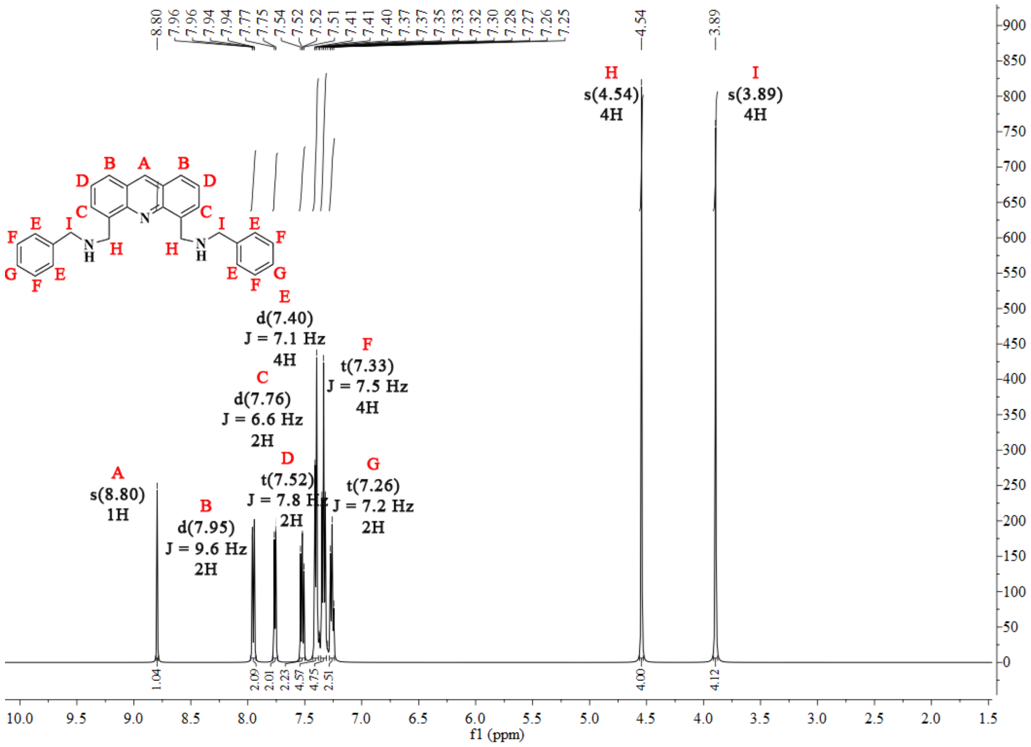

Figure S13. ^{1}H-NMR spectrum of compound **11**

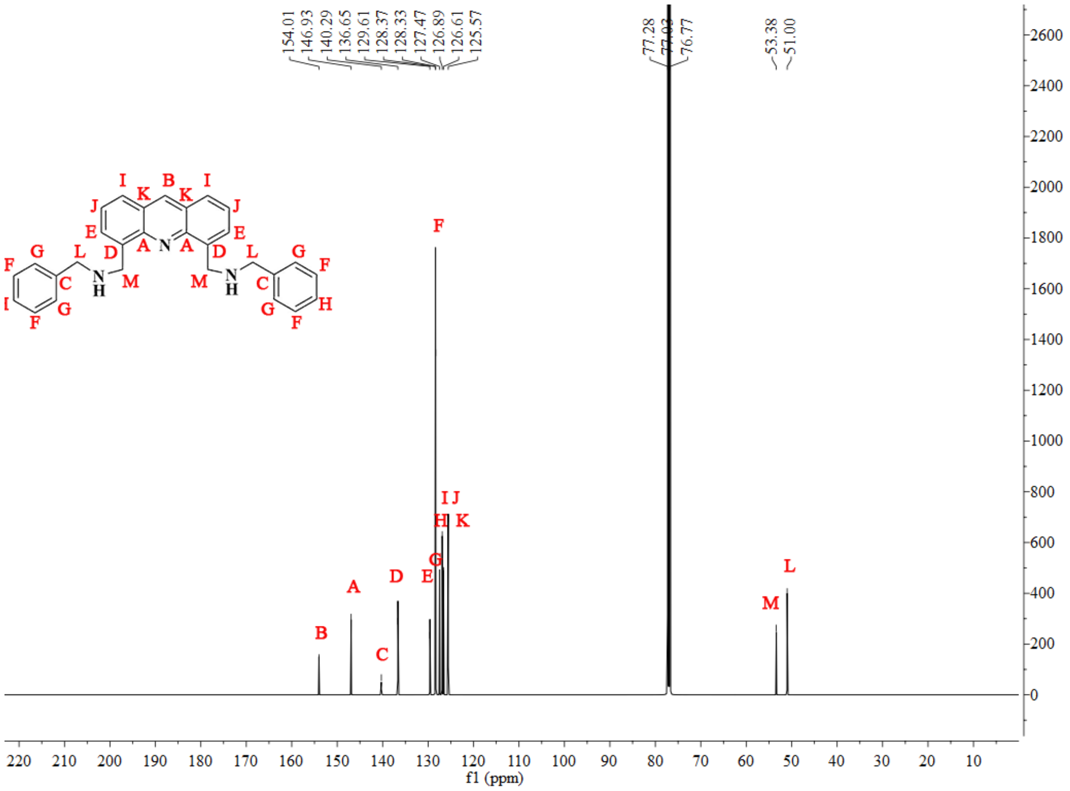

Figure S14. ^{13}C-NMR spectrum of compound **11**

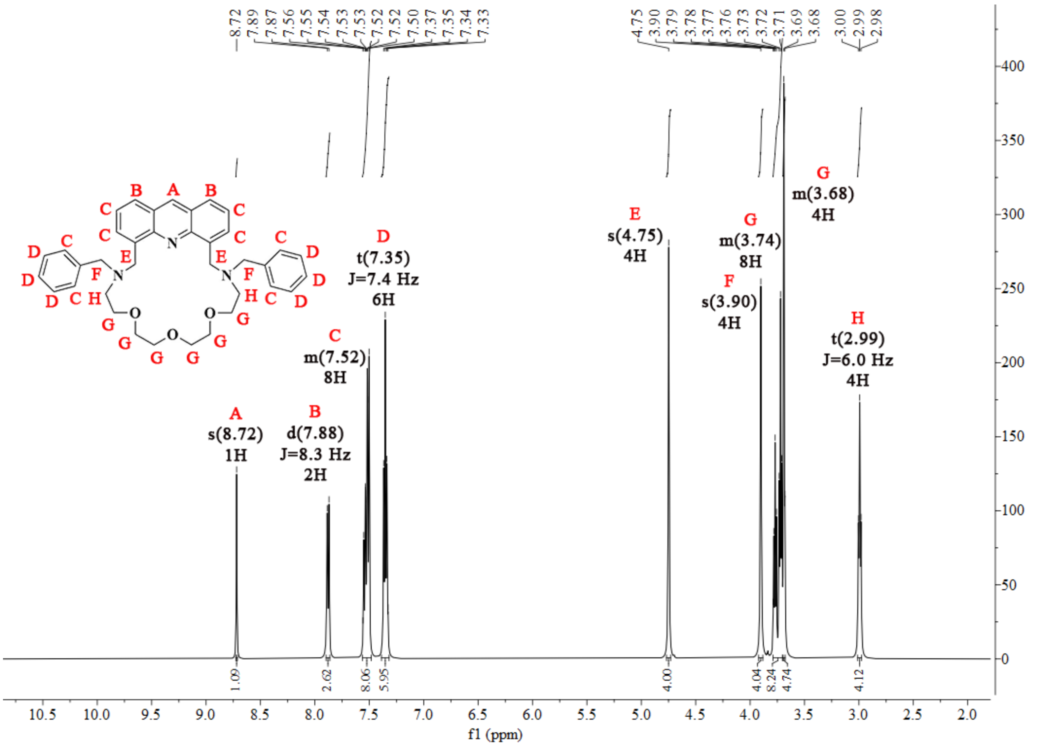

Figure S15. ^{1}H-NMR spectrum of compound **12**

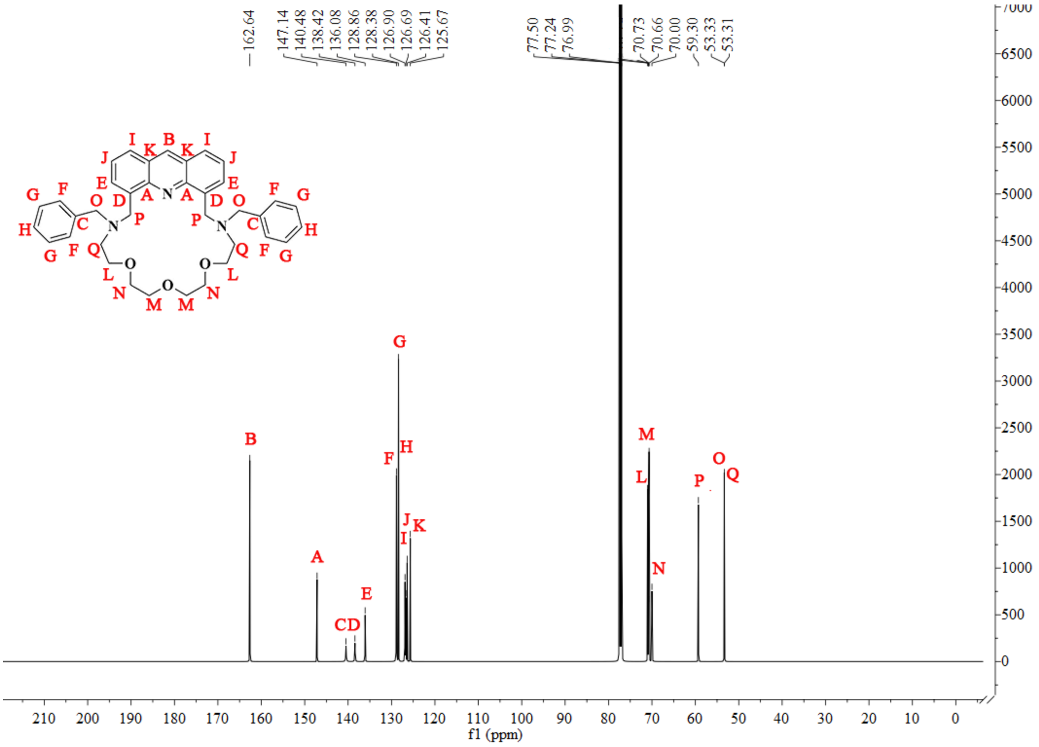

Figure S16. ^{13}C-NMR spectrum of compound **12**

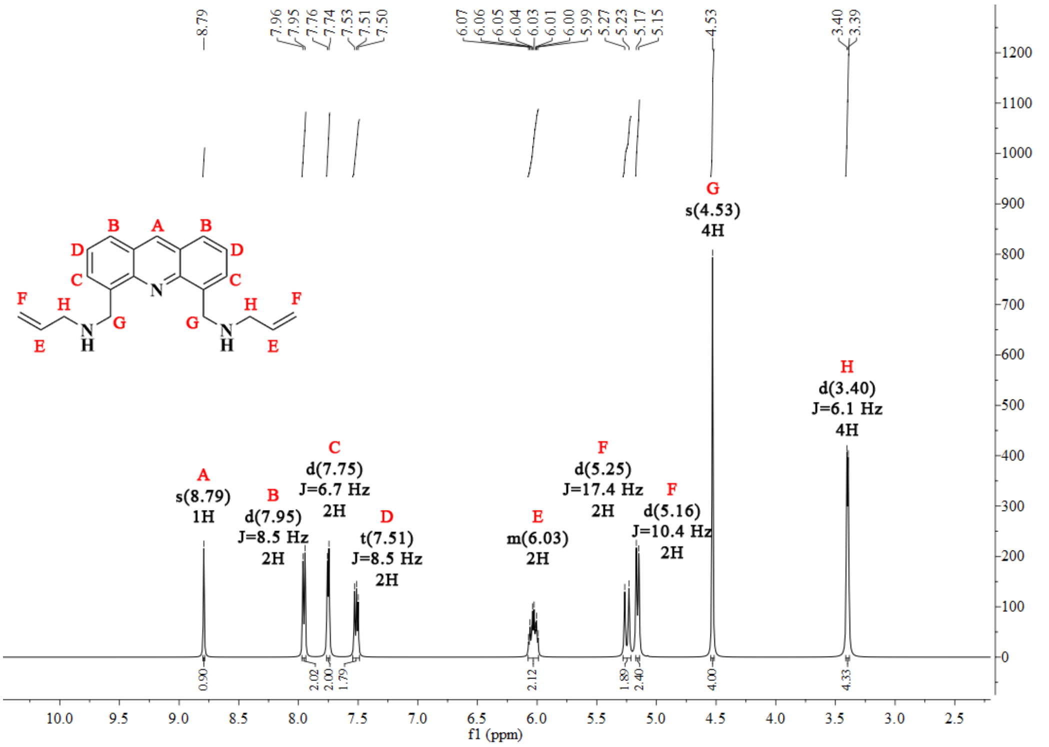

Figure S17. ^{1}H-NMR spectrum of compound **14**

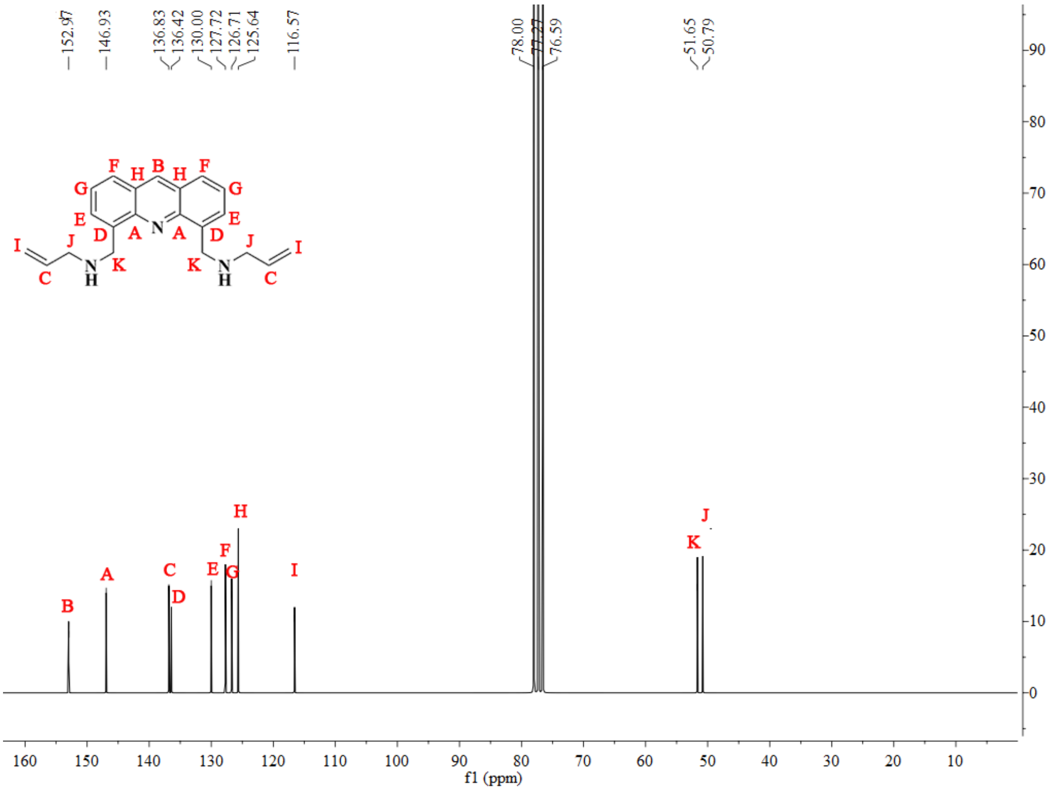

Figure S18. ^{13}C-NMR spectrum of compound **14**

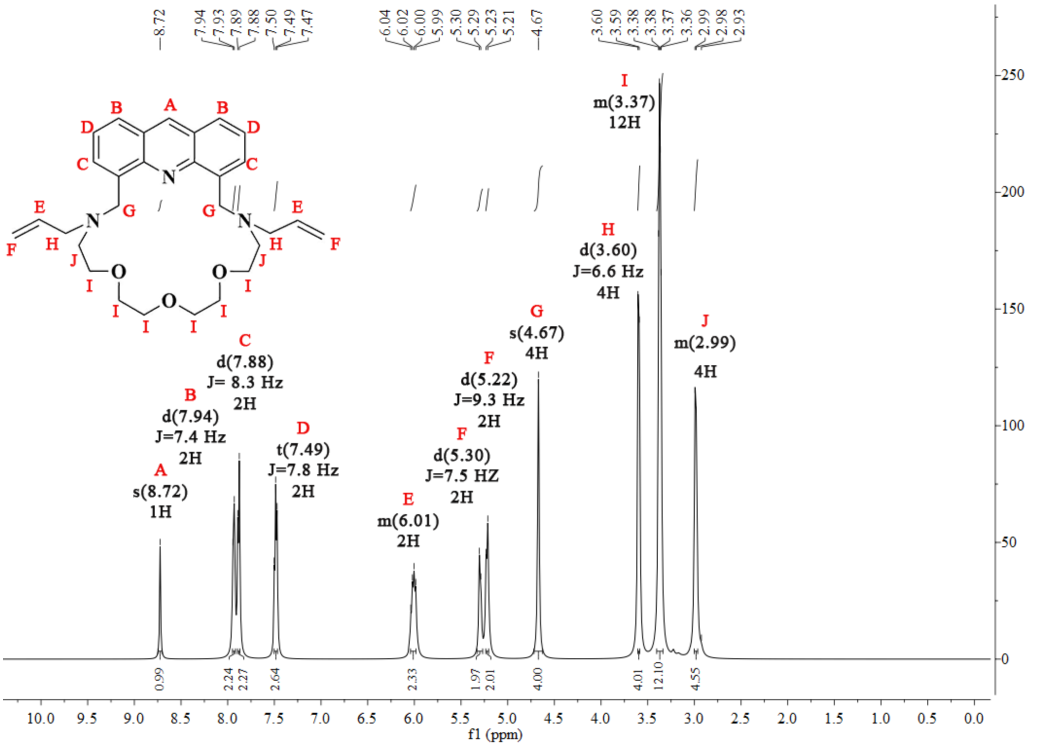

Figure S19. ^{1}H-NMR spectrum of compound **15**

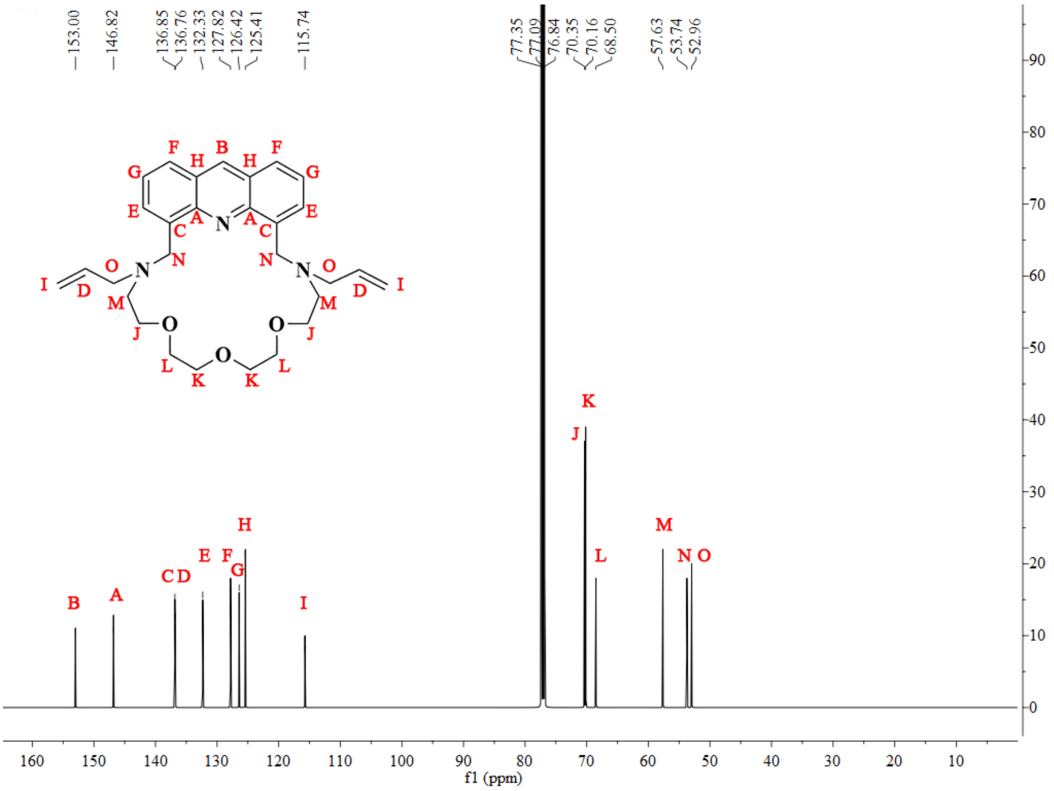

Figure S20. ^{13}C-NMR spectrum of compound **15**

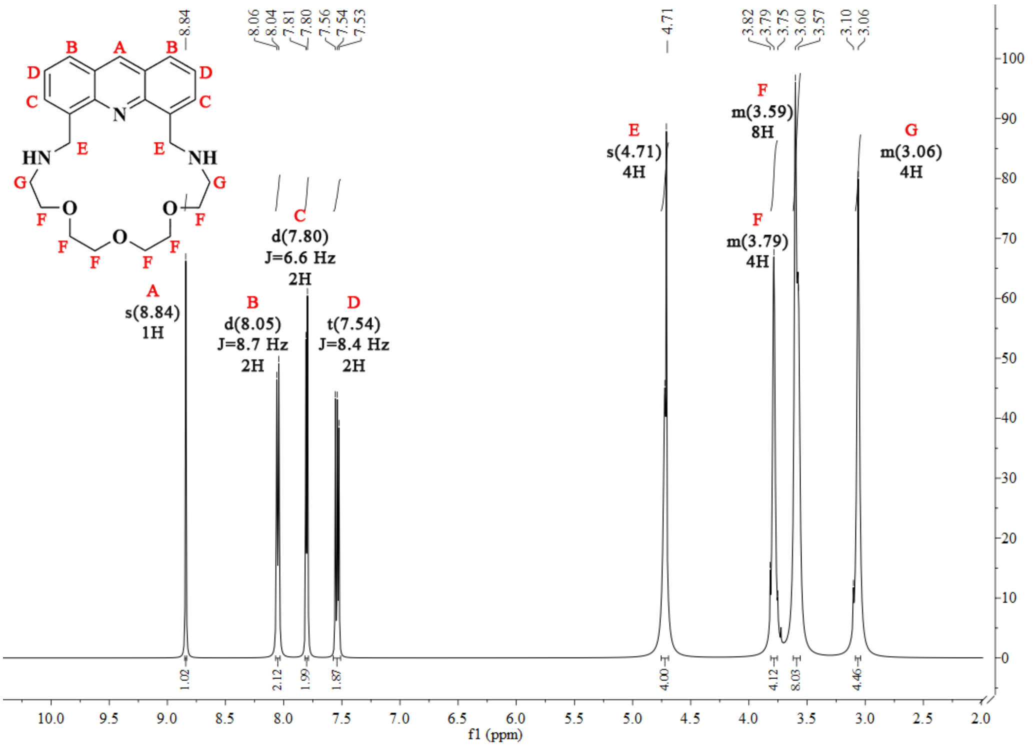

Figure S21. ^{1}H-NMR spectrum of compound **17**

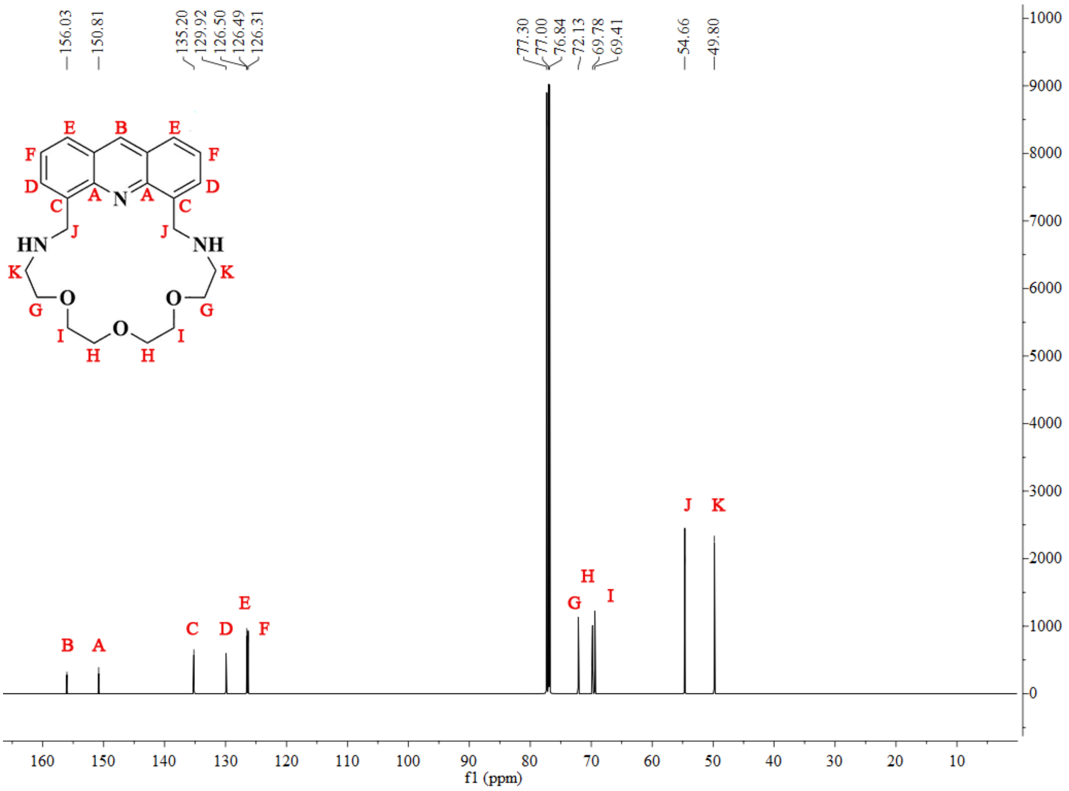

Figure S22. ^{13}C-NMR spectrum of compound **17**
